# Integration of bulk RNA sequencing and single-cell analysis reveals a global landscape of DNA damage response in the immune environment of Alzheimer’s disease

**DOI:** 10.3389/fimmu.2023.1115202

**Published:** 2023-02-21

**Authors:** Yongxing Lai, Han Lin, Manli Chen, Xin Lin, Lijuan Wu, Yinan Zhao, Fan Lin, Chunjin Lin

**Affiliations:** ^1^ Department of Geriatric Medicine, Shengli Clinical Medical College of Fujian Medical University, Fujian Provincial Hospital, Fuzhou, Fujian, China; ^2^ Fujian Provincial Center for Geriatrics, Fujian Provincial Hospital, Fuzhou, Fujian, China; ^3^ Department of Gastroenterology, Shengli Clinical Medical College of Fujian Medical University, Fujian Provincial Hospital, Fuzhou, Fujian, China; ^4^ Department of Neurology, Fujian Medical University Union Hospital, Fuzhou, Fujian, China

**Keywords:** DNA damage response, single-cell, Alzheimer’s disease, molecular subtypes, machine learning, immunity

## Abstract

**Background:**

We developed a novel system for quantifying DNA damage response (DDR) to help diagnose and predict the risk of Alzheimer’s disease (AD).

**Methods:**

We thoroughly estimated the DDR patterns in AD patients Using 179 DDR regulators. Single-cell techniques were conducted to validate the DDR levels and intercellular communications in cognitively impaired patients. The consensus clustering algorithm was utilized to group 167 AD patients into diverse subgroups after a WGCNA approach was employed to discover DDR-related lncRNAs. The distinctions between the categories in terms of clinical characteristics, DDR levels, biological behaviors, and immunological characteristics were evaluated. For the purpose of choosing distinctive lncRNAs associated with DDR, four machine learning algorithms, including LASSO, SVM-RFE, RF, and XGBoost, were utilized. A risk model was established based on the characteristic lncRNAs.

**Results:**

The progression of AD was highly correlated with DDR levels. Single-cell studies confirmed that DDR activity was lower in cognitively impaired patients and was mainly enriched in T cells and B cells. DDR-related lncRNAs were discovered based on gene expression, and two different heterogeneous subtypes (C1 and C2) were identified. DDR C1 belonged to the non-immune phenotype, while DDR C2 was regarded as the immune phenotype. Based on various machine learning techniques, four distinctive lncRNAs associated with DDR, including FBXO30-DT, TBX2-AS1, ADAMTS9-AS2, and MEG3 were discovered. The 4-lncRNA based riskScore demonstrated acceptable efficacy in the diagnosis of AD and offered significant clinical advantages to AD patients. The riskScore ultimately divided AD patients into low- and high-risk categories. In comparison to the low-risk group, high-risk patients showed lower DDR activity, accompanied by higher levels of immune infiltration and immunological score. The prospective medications for the treatment of AD patients with low and high risk also included arachidonyltrifluoromethane and TTNPB, respectively,

**Conclusions:**

In conclusion, immunological microenvironment and disease progression in AD patients were significantly predicted by DDR-associated genes and lncRNAs. A theoretical underpinning for the individualized treatment of AD patients was provided by the suggested genetic subtypes and risk model based on DDR.

## Background

Alzheimer’s disease (AD) is currently considered the most well-known form of dementia worldwide, as evidenced by the over-accumulation of extracellular amyloid plaque and the entanglement of neurofibrillary (1). The number of people with AD is positively correlated with advanced age, with more than 50 million people affected by AD (2). It is worth noting that most AD patients exhibit a poor prognosis with a median survival time of only 5-10 years due to a lack of early diagnose and effective treatment (3). Though some FDA-approved pharmacological treatments such as donepezil, rivastigmine, galanthamine, and other drugs have been used to prevent the progression of AD in the past decades (4, 5), the heterogeneity of AD patients limits the therapeutic efficacy of these drugs (6). Distinct molecular characteristics have been reported to be the main cause of AD heterogeneity, which is also closely related to the differences in clinical outcomes (7–9). Nonetheless, the potential molecular mechanisms underlying AD heterogeneity remain largely unknown. Therefore, to guide the individualized treatment of AD patients, it is necessary to clarify the heterogeneity of AD and accurately distinguish the molecular characteristics of each patient.

Genomic instability is one of the cardinal features of AD, and the DNA damage response (DDR) exerts an important role in maintaining genome integrity (10). DDR contains several well-coordinated processes, including the detection of DNA damage, the transduction of DNA damage signals, the promotion of DNA damage repair, the activation of cell cycle checkpoints, and the initiation of apoptosis when damage is irreversible (11). Intracellular DDR mechanisms enable cells to detect and repair DNA damage, and improper repair is one of the main causes of disease development and progression, including AD (12–14). Recent studies have shown that the accumulation of DNA damage is a well-recognized factor in aging and plays a vital role in the initiation of AD. It was found that DDR deficiency caused by mutations in DDR regulators, including BRAC1 occurs in the brain regions of AD and is implicated in the development of pathology (13). In addition, the breast cancer susceptibility gene 1, a DDR-associated gene, was found to accumulate in neurofibrillary tangles in AD brain. Its dysregulation is positively correlated with the pathogenesis of tauopathies (15, 16). DDR-related genes have been extensively studied in non-neural cancer tissues, but less is known in the nervous system. Therefore, it is imperative to comprehensively elucidate the expression patterns of DDR-related regulators and the potential molecular mechanisms of DDR in AD pathogenesis.

Long non-coding RNAs (LncRNAs) are a subclass of RNA molecules that are more than 200 nucleotides in length and are not translated into proteins. They are thought to be closely related to transcription, epigenetics, and post-transcriptional regulation (17). An increasing number of lncRNAs have been demonstrated to be participated in AD development and pathogenesis, and have been shown to serve as novel biomarkers for early diagnosis and effective therapeutic targets for patients with AD (18, 19). Several researchers demonstrated that lncRNAs also exert a protective role in promoting cell survival and preventing the development of various diseases *via* sustaining genomic stability. For example, as the upstream regulator of DDR, the lncRNA exerts a vital role in resisting heart failure *via* inhibiting the ataxia telangiectasia mutated (ATM)-DDR signaling pathway and increasing the activation of mitochondrial bioenergetics (20). Moreover, in the nervous system, the interaction of brain specific DNA damage-related lncRNA1 (BS-DRL1) with the chromatin protein HMGB1 induced by DDR can improve motor function and delay the degeneration of Purkinje cells in mice (21). In addition, another study also demonstrated that LncRNA Meg3 can function as the stabilizer of the DDR-related gene PTBP3 and participate in the maintenance of endothelial function (22). However, the role of lncRNA-mediated DDR signaling pathway in AD remains unknown and needs further exploration.

We thoroughly assessed the DDR expressional patterns in AD patients in our investigation. Data from single cells were used to visualize the DDR landscape of different cell types in AD. Weighted gene coexpression network analysis (WGCNA) was used to identify DDR-associated lncRNAs, and 154 AD patients were then classified into heterogeneous subtypes based on their clinical traits, biological behaviors, DDR levels, and immunological characteristics. The suitable lncRNAs connected to DDR were then chosen using four machine learning techniques, including least absolute shrinkage and selection operator (LASSO), support vector machine-recursive feature elimination (SVM-RFE), random forest (RF), and eXtreme Gradient Boosting (XGBoost). For AD patients at various risk levels, a scoring system was developed to determine their biological traits, immunological microenvironment, and prospective treatment medications. Overall, this research creatively clarified the link between DDR expression patterns and AD heterogeneity, offering novel perspectives on how to treat AD patients on an individual basis.

## Materials

### Bulk transcriptome data acquisition and pre-processing

The bulk AD transcriptome data were retrieved from the GEO (Gene Expression Omnibus, https://www.ncbi.nlm.nih.gov/geo/) database. GSE48350, GSE5281, and GSE28146 with lncRNAs and mRNAs data are based on the GPL570 platform, which included 173 normal and 80 AD brain tissue samples, 74 healthy brain tissues and 87 brain tissues from AD patients, 8 no-AD and 22 AD brain tissue samples, respectively (23–25). GSE122063 that mainly contained mRNAs data is based on the GPL16699 platform and included 44 normal and 56 AD brain tissues samples (26). In addition, we extracted mRNAs expressional data from 157 normal and 319 AD brain tissues samples from the GSE33000 dataset (built on the GPL4372 platform) (27). Since GSE48350 and GSE5281 datasets were combined based on the Combat function of “sva” R package (http://bioconductor.org/help/search/index.html?q=sva/) and a total of 8 abnormally expressed samples were removed (28). In addition, due to the high proportion of non-elderly samples identified in the normal group of the GSE48350 dataset, we chose the normal samples aged over 65 years for the further study. Eventually, a total of 150 normal and 161 AD brain tissues samples were obtained. While other three datasets GSE28146, GSE122063, and GSE33000 were selected as the validation sets. The raw data were log2-transformed and normalized according to the Robust Multiple Array Average (RMA) function of the “affy” R package http://www.bioconductor.org/help/search/index.html?q=affy/). Differential analysis was performed using the “limma” R package (http://www.bioconductor.org/help/search/index.html?q=limma/) and adjusted p-values (FDR) for DElncRNAs were determined. Genes with the value of |log2FC|>0.5 and FDR <0.05 between DDR subtypes or risk groups were determined as Differential expressed gene (DEGs).

### Single-cell sequencing data processing

The single-cell transcriptome data (15 mild cognitive impairment (MCI)/AD and 44 normal CSF samples) were obtained from the GEO database (GSE200164). The expression matrix was normalized by the “NormalizeData” function of the “Seurat” package https://cran.r-project.org/web/packages/Seurat/index.html). Integrated datasets and batch elimination were generated with the IntegrateData function of the “Seurat” package. Subsequently, the combined object underwent principal component analysis (PCA) and uniform manifold approximation and projection (UMAP) analysis. The filtering of the cells was performed based on the following parameters: Cell count >3 cells, 200 genes <Cells with <5000 genes, and cells with fewer than 15% mitochondrial genes. A total of 34738 filtered cells were selected for further analysis. The top 4000 variably expressed genes were determined by the “FindVariableFeatures” function of the “Seurat” package. The “FindClusters” function was utilized to classify the cells into distinct clusters. Cell type annotation was performed based on the “Celltypist” Python package according to a prior study (29). Additionally, DEGs for each cell cluster were identified by utilizing the “FindAllMarkers” function with logfc.threshold = 0.25.

### Establishment of a DDR score

A total of 200 DDR regulators were extracted according to previous studies (30, 31). Differentially expressed DDR regulators were visualized using a heatmap, and correlations between these DDR regulators were visualized using a gene network diagram drawn from the igraph package. Furthermore, a DDR score were estimated based on the differentially expressed DDR regulators *via* the Single-Sample Gene Set Enrichment Analysis (ssGSEA) or “Ucell” algorithms (32). Spearman described a correlation analysis between DDR scores and 28 infiltrated immune cells.

### Cell communication analysis

The CellChat objects were created by the “CellChat” R package (https://www.github.com/sqjin/CellChat) (33) based on the UMI count matrix for each group (Normal and AD). The “CellChatDB.human” ligand-receptor interaction database was considered preference data. Cell-to-cell communication analysis was conducted using the default parameters set. CellChat objects from each group were combined using the “mergeCellChat” function to obtain a comparison of the total number of interactions and the strength of interactions. The visualization of the differences in the number or strength of interactions among distinct cell types between groups was achieved using the “netVisual_diffInteraction” function. Finally, we determined differential expression of signaling pathways using the “rankNet” function and visualized the distribution of signaling gene expression between groups using the “netVisual_bubble” and “netVisual_aggregate” functions.

### Investigation of DDR-associated lncRNAs

The weighted gene coexpression network analysis (WGCNA) approach was employed to examine lncRNA modules associated with DDR score *via* the “WGCNA” R package (https://cran.rstudio.com/web/packages/WGCNA/) (34). Briefly, the expression landscapes of lncRNAs were selected as input data and converted into adjacency matrix to further establish unsupervised co-expression association. According to the scale-free topology criteria, the scale-free network was built based on the optimal soft threshold identified, followed by the constructed weighted adjacency matrix converted into a topological overlap matrix (TOM). Then, we employed a dynamic tree pruning algorithm to identify lncRNA modules of different colors in the hierarchical clustering tree. A LncRNA module that displayed the strongest correlation with the DDR score was finally screened and selected for further analysis.

### Consensus clustering based on differentially expressed DDR

DDR-associated lncRNAs shared by the turquoise module, combined datasets, and GSE5281 were identified as AD-related lncRNAs, and DDR subtypes were developed using the k-means method implemented in the “ConsensusClusterPlus” R package (http://www.bioconductor.org/help/search/index.html?q=ConsensusClusterPlus/) (35) with the following parameters: resampling rate per iteration=80%, cluster classification = 2–8, repetitions = 1000, and Euclidean distance. The appropriate number of DDR subtypes was determined on the basis of the consensus score matrix, cumulative distribution function (CDF) curve, the relative change in the area of the CDF curve, and the consistency of the cluster score. The t-Distributed Stochastic Neighbor Embedding (tSNE) plot was applied for evaluating the stability of the clustering patterns.

### Enrichment analysis and functional annotation

Kyoto Encyclopedia of Genes and Genomes (KEGG) Gene ontology (GO) enrichment analysis were performed using the previously described “clusterProfiler” R package (http://www.bioconductor.org/help/search/index.html?q=clusterProfiler/) (36). Gene ontology biological function, including biological processes (BP), molecular function (MF), and cellular component (CC). The p-values ​​were adjusted using the Benjamini-Hochberg method, and the adjusted p-values below 0.05 were considered statistically significant.

Gene Set Variation Analysis (GSVA) enrichment was conducted to evaluate the heterogeneity of a variety of biological processes and pathway activities using the “GSVA” R package (http://www.bioconductor.org/help/search/index.html?q=GSVA/) (37). Hallmark gene sets of “c2.cp.kegg.v7.4.symbols” and “c5.go.bp.v7.5.1.symbols” obtained from the Molecular Signatures Database (MSigDB, http://www.gsea-msigdb.org/gsea/msigdb/index.jsp ) were chosen as the preference gene sets of GSVA. Different molecular features were elucidated in DDR subtypes. Differences between biological functions and signaling pathways were calculated using the “limma” R package, and absolute t-values ​​with a GSEA score greater than 5 were considered statistically significant.

Furthermore, we performed gene set enrichment analysis (GSEA) based on the “clusterProfiler” R package to assess the differences in pathway activities. Normalized enrichment score (NES) were ranked, and adjusted p-values below 0.05 were considered statistically significant.

### Evaluation of the immune microenvironment

The immune infiltrating levels were conducted using ssGSEA https://github.com/GSEA-MSigDB/ssGSEA-gpmodule), MCPcounter https://github.com/ebecht/MCPcounter), xCell https://xcell.ucsf.edu/), ABIS https://github.com/giannimonaco/ABIS) and ESTIMATE (https://cibersortx.stanford.edu/) algorithms according to previously described (38). Briefly, the proportions of a variety of immune cells in each sample were assessed using global marker genes, and the above algorithms were applied for calculating fractional enrichment or relative abundance for each immune cell subset. The Wilcoxon rank-sum test was employed to evaluate the differences in immune infiltration levels between groups. A heatmap was utilized to visualize the abundance of immune infiltration for each AD sample under distinct algorithms. Additionally, immune scores were calculated using the “ESTIMATE” R package to further estimate the immune microenvironment of patients with AD. Finally, the expression levels of 60 immunoregulatory genes such as antigen presentation, cell adhesion, co-inhibitor, co-stimulator, ligand, and receptor were described to explore the differences in immune competence between groups.

### Screening of characteristic lncRNAs based on machine learning

Four distinct machine learning algorithms, including LASSO, SVM-RFE, RF, and the XGBoost model were conducted to predict the DDR-associated characteristic lncRNAs on the basis of “glmnet” (https://cran.r-project.org/web/packages/glmnet/index.html) “e1071” (https://cran.r-project.org/web/packages/e1071/index.html), “caret” https://cran.r-project.org/web/packages/caret/index.html), “Boruta” https://cran.r-project.org/web/packages/Boruta/index.html), and “xgboost” (https://github.com/dmlc/xgboost) packages. In the LASSO model, the final coefficients of the important variables were finally determined based on the optimal penalty parameter λ through five cross-validations (39). The Boruta-based identification of significant lncRNA signatures was performed using the following parameters: (300 iterations and p-value less than 0.01). After excluding the insignificant variables, those remaining Boruta-based lncRNA signatures were fitted to the RF model by the “caret” R package and ranked according to the calculated lncRNA importance. The SHAP values were employed to interpret the XGBoost model *via* the “SHAP” (https://github.com/pablo14/shap-values) R package. These four machine learning models were conducted with default parameters based on 5-fold cross-validation to avoid over-fitting. All the brain tissue samples enrolled in the combined datasets were randomly split into a training set (70%) and a verification set (30%). The results of the four machine learning algorithms were then intersected to determine the final characteristic lncRNA. The diagnostic performance was assessed using the area under the receiver operating characteristic curve (AUC) on the basis of the “pROC” (https://cran.r-project.org/web/packages/pROC/) R package.

### Construction of a nomogram and a risk model based on DDR-related lncRNAs

The distinctive lncRNAs associated with DDR were then applied for generating a predicted nomogram based on the “rms” (https://cran.r-project.org/web/packages/rms/index.html) package. A calibration curve was made to figure out how accurate the nomogram was. Using the “ggDCA” (https://cran.r-project.org/web/packages/ggDCA/index.html) R package, the results of a decision curve analysis (DCA) were employed to measure and evaluate the nomogram’s clinical performance.

DDR riskScore were calculated using the characteristic lncRNA signature coefficients generated by the LASSO machine learning model: riskScore = Σ_i_ Coefficients_i_ × Expression level of lncRNA_i_ (i represents the each lncRNA symbol that was included in the risk scoring model). All patients with AD can be classified according to their risk score. Finally, AD samples with a risk score below or above the median were divided into the low-risk group and the high-risk group. Finally, based on the obtained DDR-related lncRNAs, a risk score model was constructed to classify 154 AD patients with high- and low-risk groups in the combined dataset. An external dataset GSE5281 was employed to verify the applicability of risk estimation.

### Prediction of potential therapeutic drugs

Connectivity map (CMap) analysis is a widely utilized approach to predict treatments for patients based on similar gene expression profiles (40). In this study, the drug signature data retrieved from the CMap database was chosen as the preference drug information, and the top 150 up-regulated and 150 down-regulated genes in AD patient with high-risk and low-risk, respectively, were selected as input data. Based on the using the eXtreme Sum (XSum) algorithm, we then compared the similarity of gene expression and drug signatures and calculated the CMap scores to assess therapeutic potential in distinct risk patients.

### Other statistical analysis

All statistical analyses and visualizations were performed using R 4.1.0 software. Spearman’s correlation analysis was used to clarify the relationship between two continuous variables. The Wilcoxon sum-rank test or t-test was used to compare the difference of continuous variables between two groups. Comparisons of non-continuous variables between the two groups were made using the chi-square test. Binary categorical variables were predicted by ROC curve analysis. FDRs were calculated according to the Benjamin-Hochberg method to adjust p-values, and a two-sided p-value below 0.05 was considered to be statistically significant.

## Results

### The role of DDR-related genes in patients with AD

**Figure 1:**
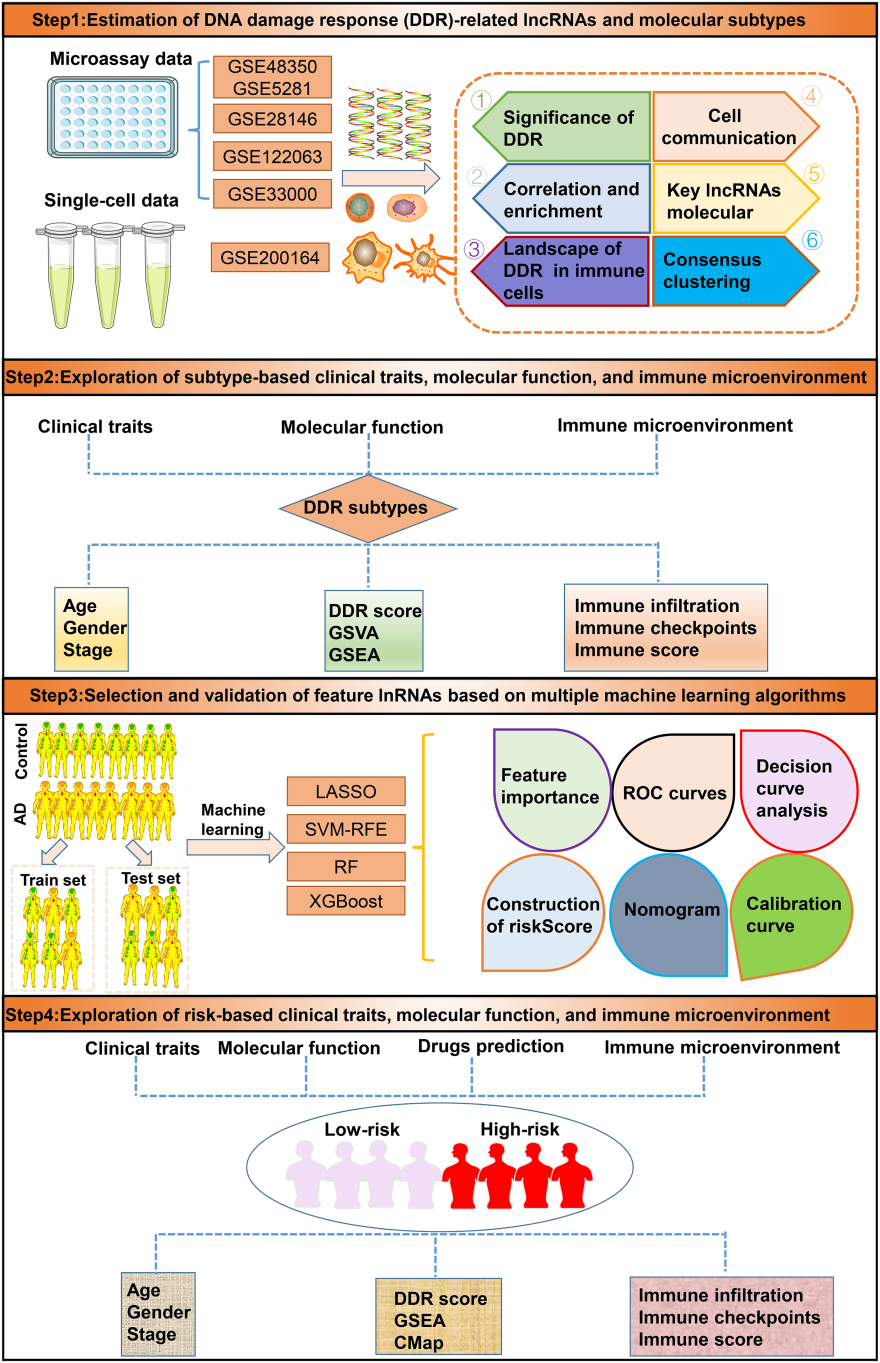
The study flow chart.

**Figure 2:**
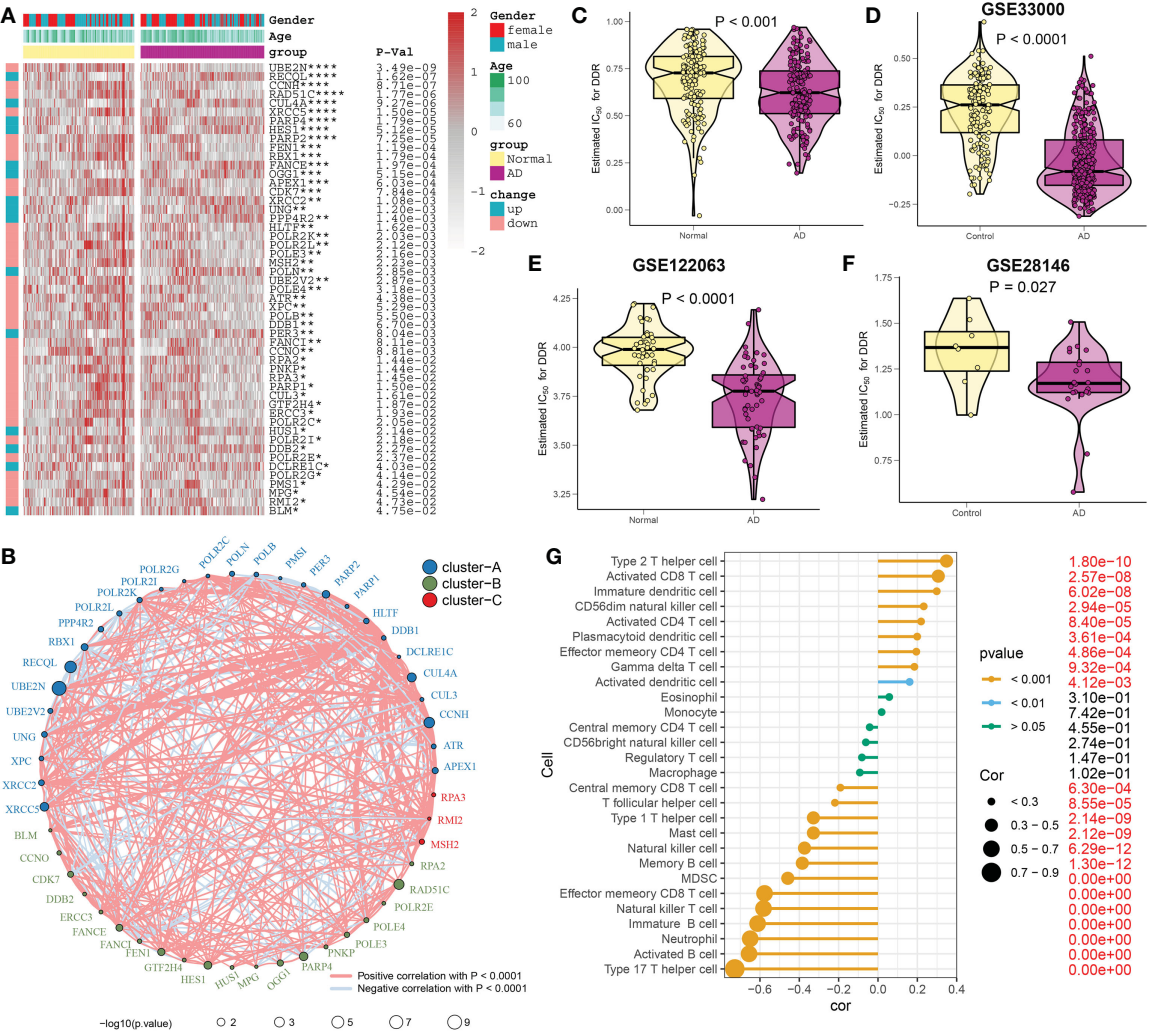
Estimation of DDR regulators between AD and healthy individuals. **(A)** Comparison of the expression profiles of abnormal DDR regulators across AD and non-AD brain tissues in combined dataset using a heatmap. As examples of annotations, age and sex were displayed. **(B)** Interactions among 51 DDR regulators. The circle size serves as a representation of how each individual variable affected AD, and the Benjamini-Hochberg method is used to modify the p-value. Different colors indicating that DDR regulators are grouped into distinct clusters. The interactions are represented by each line joining the DDR regulators, and the thickness of each line reflected the intensity of the link. Blue denotes a negative correlation, whereas red denotes a positive connection. **(C–F)** The DDR scores of AD and non-AD brain tissues are compared in the combined datasets **(C)**, GSE33000 **(D)**, GSE122063 **(E)**, and GSE28146 **(F)** respectively. **(G)** The relationship between 28 immune cell subtypes and the DDR score in combined dataset. *p < 0.05, **p < 0.01, ***p < 0.001, ****p < 0.0001.

A detailed flow chart of the research process is shown in **Figure 1**. After excluding aberrant brain tissues samples, we merged the expression patterns of the normal and AD brain tissues from the GSE48350 and GSE5281 datasets, yielding 150 normal and 161 AD brain tissues. Brain tissues from various platforms displayed considerably diverse clustering patterns before batch effects were removed, but they grouped together after batch correlation (Additional file: **Figures S1A, B**). Normal individuals had a mean age of 81.58 years (SD: 8.85), and 65 (43.3%) subjects were females. Interestingly, AD patients exhibited a mean age of 82.47 years (SD: 7.64), and 83 (49.7%) of which were females. The differences in age and sex between the normal and AD groups were not statistically significant (Additional file: **Figures S1C, D**). Of the 200 DDR-related regulatory genes obtained in previous studies, 179 genes were expressed in the AD-related combined dataset. Abnormal expression profiles of 51 DDR regulators were observed in AD brain tissues when compared with those in normal group, suggesting a marked difference in biological behavior between AD patients and healthy subjects. The heatmap displayed the expression landscape of 51 differentially expressed DDR-related genes between healthy individuals and patients with AD. Among them, RECOL, CUL4A, PARP4, HES1, FANCE, OGG1, XRCC2, UNG, PPP4R2, POLN, PER3, HUS1, DDB2, DCLRE1C, and BLM were found to be dramatically increased in AD brain tissue samples, while a significant decrease in the expression levels of other 36 genes including UBE2N, CCNH, RAD51C, XRCC5 and et al. was observed in AD group relative to normal group (**Figure 2A**). Functional annotation analysis revealed that these differentially expressed DDR regulators were primarily enriched in DNA damage repair-associated progresses, including nucleotide-excision repair, double-strand break repair, DNA recombination, transcription factor TFIIH core complex, and DNA-directed 5’-3’ RNA polymerase activity (Additional file: **Figure S1E**). In addition, KEGG analysis suggested that some human disease, genetic information processing-associated signaling pathways, cell cycle, and cytosolic DNA-sensing pathways were closely related to these DDR regulators (Additional file: **Figure S1F**). To systemically elucidate the relationship between the 51 differentially expressed DDR regulators, we grouped these genes into three types and generated a regulatory network showing the interactions among these DDR-related genes, most of which showed substantial associations. For example, UBE2N and PARP2, also from cluster A, presented a significant synergistic effect (coefficient = 0.645), whereas the same cluster B-associated DDR regulators, such as HES1 andRAD51C, exhibited a highly antagonistic effect (coefficient = -0.418). Additionally, Furthermore, a strongest positive correlation was detected between PARP2 and UBE2V2 (coefficient = 0.738). Conversely, PARP2 and OGG1 possessed the dramatic negative correlation (coefficient = -0.513) (**Figure 2B**). Subsequently, to further illustrate the relationship between DDR and AD, we calculated the DDR score for each sample using the ssGSEA algorithm based on the differentially expressed DDR regulators. Interestingly, we found AD patients exhibited significantly lower DDR score relative to non-AD individuals in the combined dataset (**Figure 2C**), which was consistent with the results from the other three external validation datasets, GSE33000, GSE122063, and GSE28146 (**Figures 2D–F**). These results indicated that the interactions among these DDR-regulated genes may play a major role in preserving genomic integrity, while the dysregulation of DDR may be the vital factor leading to AD progression. Furthermore, we further described the correlation patterns between DDR and 28 infiltrated immune cell subsets (**Figure 2G**). The lower DDR score represented a superior levels of immune cell infiltration, suggesting the synergistic effects of low DDR scores and highly infiltrated immune cells may promote the progression of AD.

### Analysis of DDR at the level of single-cell

**Figure 3:**
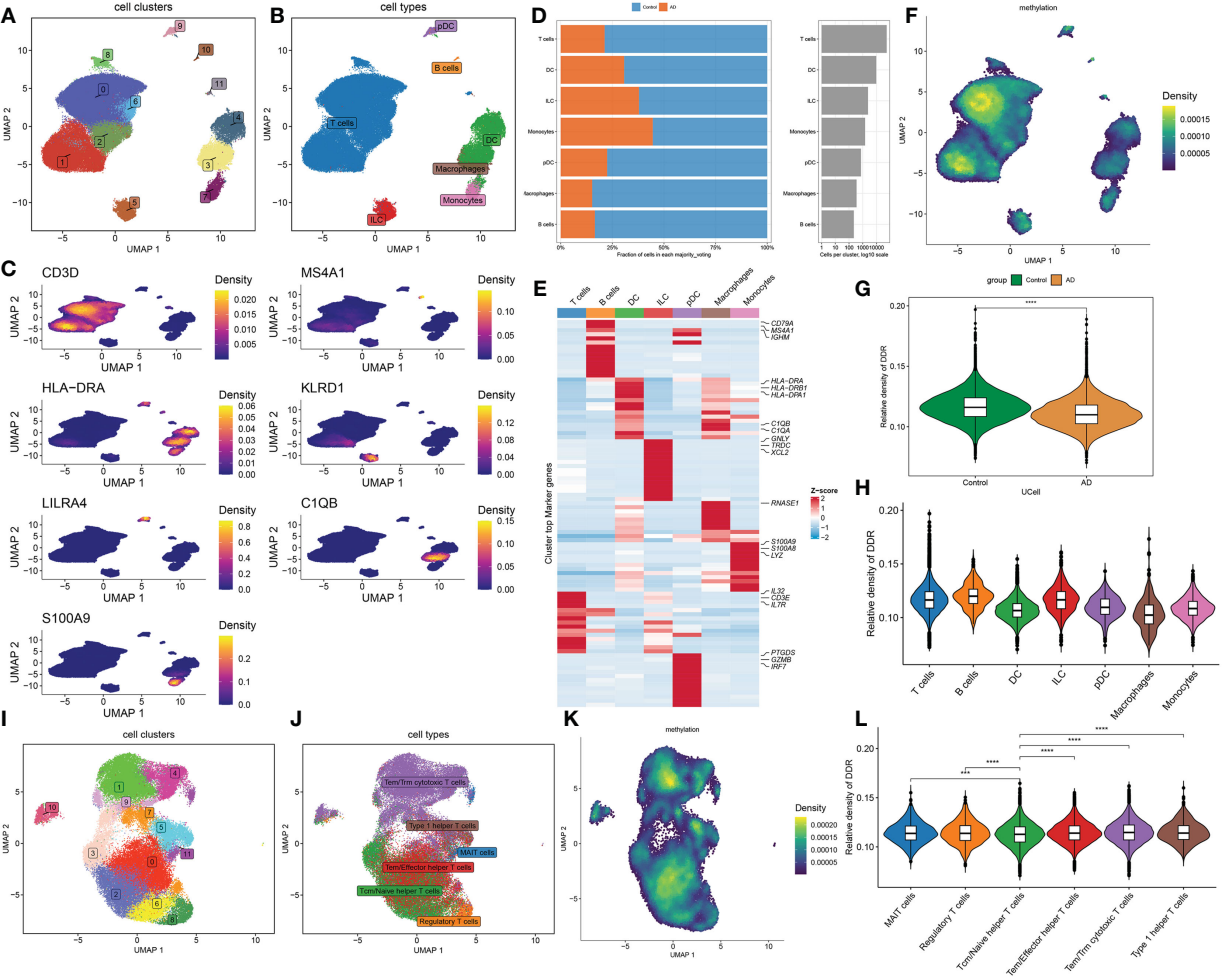
Characteristic of DDR at the single-cell level. **(A, B)** The UMAP projection of 70391 single cells from 15 MCI/AD patients and 44 control subjects, showing the formation of 12 main clusters **(A)**, which were further annotated as seven cell types, including T cells, B cells, DC, ILC, pDC, macrophages, monocytes **(B)**. Each dot corresponds to one single cell, colored according to cell cluster. **(C)** Representative UMAP plots showing the expression levels of marker genes representing seven cell subtypes in 70391 cells from both normal and cognitively impaired CSF samples. **(D)** A stacked bar chart showing the fractions of each cell type in normal and CI groups, respectively. **(E)** A heatmap displaying the distribution of top 15 differentially expressed genes specific to different cell subtypes. Blue colors denote down-regulation, whereas red colors denote up-regulation. **(F)** Difference of DDR score based on the UCell algorithm across 70391 single cells were illustrated using UMAP plots. **(G)** Representative violin plots displaying the differences in DDR score between control and CI groups. **(H)** Representative violin plots displaying the differences in DDR score among seven distinct cell types. **(I, J)** UMAP plot showing clusters **(I)** and annotated subtypes **(J)** of CSF T cells. **(K)** UMAP plot exhibiting the distribution of DDR score based on the UCell algorithm across distinct T cell subtypes. **(L)** Representative violin plots exhibiting the differences in DDR score among six distinct T cell subtypes. ***p < 0.001, ****p < 0.0001.

To investigate the differences in DDR activity of various infiltrated immune cells in AD, we performed an in-depth analysis of public single-cell sequencing data related to cognitive impairment. A total of 70391 sorted cells from 15 MCI/AD and 44 normal CSF samples were grouped into 12 clusters, and seven cell types were finally annotated, including T cells (n=55435) identified by the expression of CD3D, B cells (n=224), marked by MS4A1, DC (n=9749) which expressed the HLA-DRA, ILC (n=2406) marked by KLRD1, pDC (n=738) identified by LILRA4, macrophages (n=354) which were positive for C1QB, monocytes (n=1485) defined by their classical marker S100A9 (**Figures 3A–C**). Cell type fractions of each group revealed a decreased percentage of all cell types in the cognitive impairment (CI) group (**Figure 3D**). The heatmap exhibited the expression patterns of top 15 marker genes for each cell subtype (**Figure 3E**). We then calculated the DDR score of each cell using the “UCell” algorithm, the results suggested that cells with a higher DDR score were primary enriched in normal samples, especially in the regions of T and B cells (**Figures 3F–H**), which was consistent with the results of bulk transcriptomic analysis.

Since T cells accounted for the largest proportion, we next further explored the expression of DDR in distinct T cell subsets. The T cells were extracted and reanalyzed, and a total of 12 clusters were identified, which were annotated as six T cell subtypes, including 761 MAIT cells identified by SLC4A10, 1779 regulatory T cells which were represented as FOXP3, 13904 Tcm/Naive helper T cells marked by CCR7, 14876 Tem/Effector helper T cells defined by TIMP1, 22822 Tem/Trm cytotoxic T cells marked by GZMK, and 1132 Type 1 helper T cells marked by CXCR6 (**Figures 3I, J**, Additional file, **Figure S2A**). All six major cell types were presented significant difference between control and CI samples (Additional file, **Figure S2B**). The UCell algorithm results revealed that DDR scores were upregulated to varying degrees in all T subtypes except Tcm/Naive helper T cells (**Figures 3K, L**).

### Cell–cell interactions within the progression of cognitive impairment

**Figure 4:**
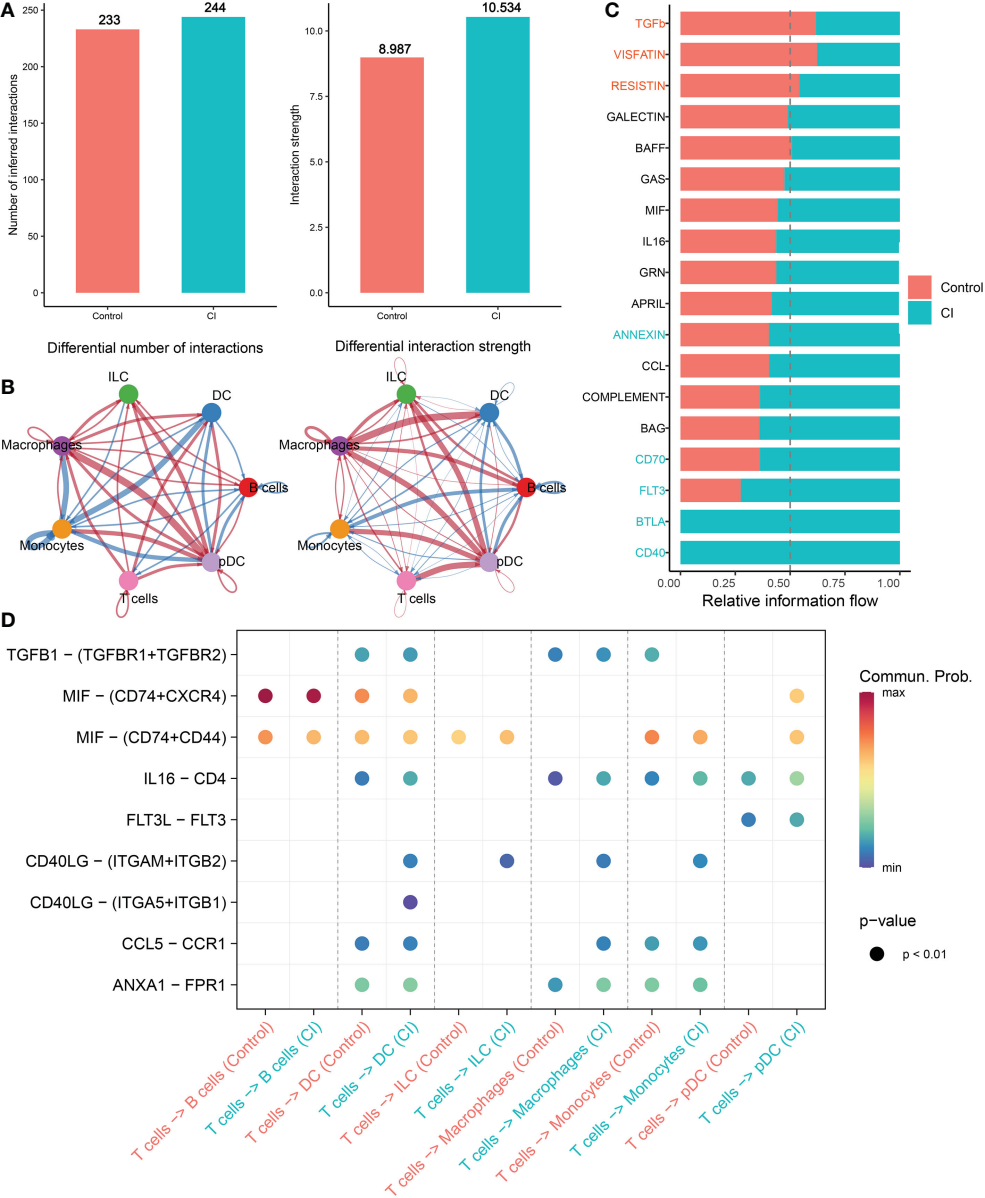
Intercellular communication difference between Normal and AD single cells**. (A, B)** Bar and circle charts showing the differences in the number of interactions (left) or strength of interactions (right) in the network of cell-cell communication between normal and CI groups. Thicker lines representing stronger interactions, and red or blue colors representing greater or decreased signaling in AD patients when compared with normal group, respectively. **(C)** Stacked plots exhibiting the differences in intercellular signaling pathways between CI and normal groups. Orange and green colors denote up-regulated signaling pathways in normal and CI samples, respectively. **(D)** Dot plot indicating the difference in signaling molecules from T cells to other immune cells between normal and cognitively impaired CSF samples.

Subsequently, we conducted the CellChat analysis to explore cell-cell interactions during the progression of cognitive impairment. The interaction numbers and strength strengthened from normal to cognitive impairment. In particular, macrophages, ILC, and pDC cells exhibited stronger interaction numbers and interaction strengths with other cell types in the CI group relative to the normal group. While DC cells and B cells communicated frequently with monocytes and pDC cells in the normal group (**Figures 4A, B**). Specific pathways were then identified between the control and CI groups by comparing the interaction strengths of each pathway. Signaling pathways such as ANNEXIN, CD70, FLT3, BTLA, and CD40 were particularly active in cognitively impaired patients. However, the control individuals exhibited a relative greater activity of TGFb, VISFATIN, and RESISTIN signaling pathways. For example, the impairment of the TGFb and ANNEXIN signaling pathway in CI patients was primarily reflected in the reduction of monocytes (senders) and DC cells (receivers) and ILC (receivers) interactions, while the frequent communication between B cells (senders) and T cells (receivers) lead to the increment of the CD70 signaling pathway in CI group (**Figure 4C**, Additional file: **Figures S2C–H**). In addition, the strong communication probabilities between T cells and other cell types, including DC, ILC, macrophages, and pDC binding CD4 and FPR1 IL16 *via* ANXA1were observed in cognitively impaired CSF samples. However, the communication between CD40LG and ITGAM+ITGB2 and ITGA5+IRGB1 was unique to CI group (**Figure 4D**).

### Investigation of DDR-associated long non-coding RNAs

**Figure 5:**
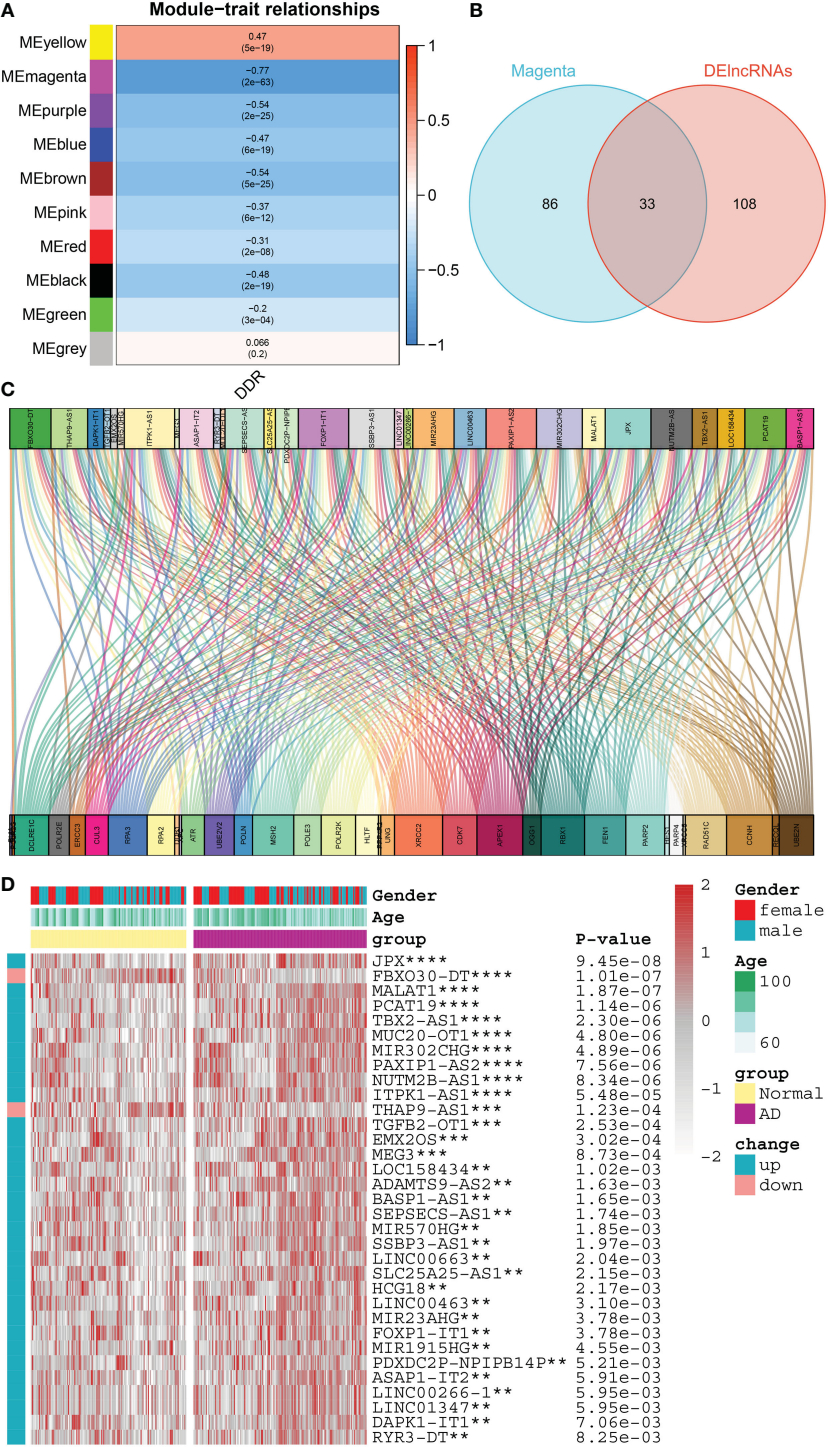
Identification of DDR-associated lncRNAs. **(A)** Heatmap representing the association between 10 modules with DDR score. **(B)** The intersecting DDR-associated lncRNAs shared by the magenta module and the DElncRNAs in the combined dataset were shown in a venn diagram. **(C)** The Sankey diagram exhibiting the correlation between differentially expressed DDR regulators and 33 lncRNAs. **(D)** Comparison of the expression profiles of 33 DDR-associated lncRNAs across AD and normal brain tissues in combined dataset using a heatmap. **p < 0.01, ***p < 0.001, ****p < 0.0001.

To further elucidate the regulatory patterns of DDR, we conducted the WGCNA approach and screened DDR-associated lncRNAs on the basis of the expression profiles of lncRNAs in the combined dataset. Next, according to the optimal soft threshold β, we applied a hierarchical clustering algorithm to the samples of the clustering dendrogram, and thus obtained ten lncRNA co-expression modules with different colors. Among them, the magenta module exhibited the most powerful link with DDR score (R= -0.77) (**Figure 5A**). Following intersection, 33 common DDR-associated lncRNAs were finally screened (**Figure 5B**). The DDR-lncRNA co-expression network generated is visualized in **Figure 5C**. The heatmap exhibited the diverse expression patterns of 33 DDR-associated lncRNAs between controls and patients with AD, of which 31 were found to be markedly reinforced in AD patients, whereas the expression profiles of the other 2 DDR-associated lncRNAs were observed to be substantially enhanced in healthy individuals (**Figure 5D**).

### Identification of DDR subtypes in AD patients

**Figure 6:**
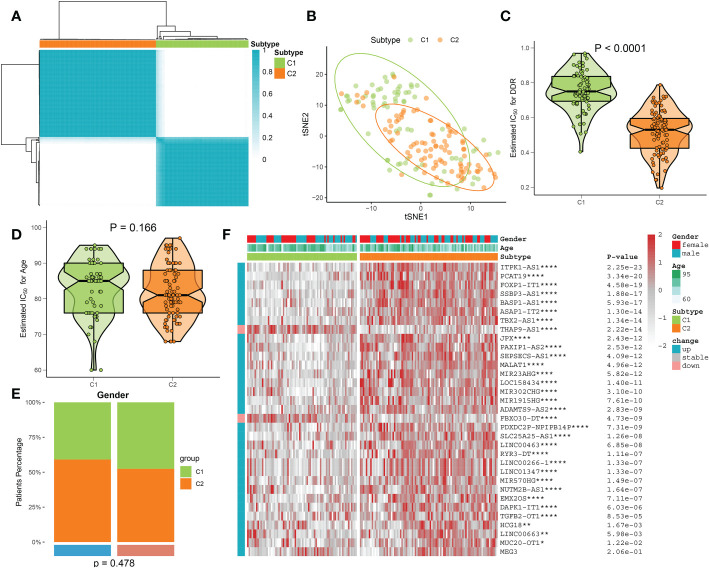
Identification of DDR subtypes using a consensus clustering algorithm. **(A)** The consensus clustering matrix of DDR subtypes is depicted based on the consensus clustering methodology. **(B)** The DDR C1 and C2 samples are differentiated using the t-SNE diagram. **(C)** A comparison of the DDR score in AD patients with DDR C1 and C2. **(D, E)** Age **(D)** and gender **(E)** distribution differences between AD patients with DDR C1 and C2. **(F)** Comparison of the expression profiles of 33 DDR-associated lncRNAs across DDR C1 and C2 subtypes in AD patients using a heatmap. *p < 0.05, **p < 0.01, ****p < 0.0001.

On the basis of the expression landscape of 33 DDR-related lncRNAs, a consensus clustering algorithm was used to classify AD samples into different subtypes, each with a qualitatively different DDR regulatory status. The higher the consensus matrix score, the more likely it was to be in the same group (**Figure 6A**). In addition, the smoother the center of the CDF curve, the clearer the sampling distribution. Ultimately, combining the relative changes in the area under the CDF curve and consistent clustering score results, a total of 167 AD patients were grouped into two subtypes, including 74 cases of C1 and 93 cases of C2 (Additional file: **Figure S3**). The tSNE analysis revealed significant differences in the distribution of AD patients between the C1 and C2 clusters (**Figure 6B**). Interestingly, DDR C2 showed a lower DDR score relative to DDR C1 (**Figure 6C**), suggesting a lack of DDR regulation in patients with DDR C2. However, the age and gender distribution were not statistically different between DDR C1 and DDR C2 subtypes (**Figures 6D, E**). A heatmap illustrated that these DDR-related lncRNAs were notably different between distinct DDR clusters, and most lncRNAs were upregulated in DDR C2 (**Figure 6F**).

### Characterization of the molecular functions and pathways between DDR subtypes

**Figure 7:**
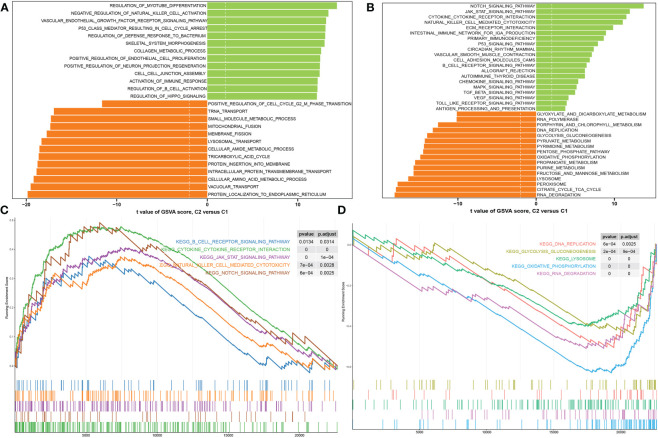
Analyses of functional enrichment in DDR subtypes. **(A, B)** The t-value of the GSVA scores is utilized to rank the differences in abundant biological functions **(A)** and characteristic signaling pathways **(B)** between DDR C1 and C2. **(C, D)** GSEA revealing the elevated **(C)** and downregulated **(D)** main pathways in patients with DDR C2.

We next attempted to illustrate the transcriptomic differences between these DDR subtypes using the DEG analysis, and a total of 2472 DEGs between these subtypes were screened (2859 up-regulation and 1856 down-regulation) (Additional file: **Figure S4**). Functional annotations based on the GSVA algorithm revealed that DDR C2 was mainly involved in myotube differentiation, activation of vascular endothelial growth factor receptor, intercellular junctions, neuron projection regeneration, cell cycle arrest, and immune responses. In contrast, DDR C1 was closely linked with the biological functions associated with mitochondrial and amino acid metabolism, tRNA and lysosomal transport, and protein localization (**Figure 7A**). Pathways enrichment analysis showed that DDR C2 was primarily driven by immune regulation, cytokine-cytokine interaction, and several classical signaling pathways, including JAK-STAT, P53, TGFβ, MAPK, and VEGF signaling pathway. Notably, DDR C1 predominantly regulated enriched pathways associated with metabolism, DNA replication, lysosome, gluconeogenesis, and RNA degradation (**Figure 7B**). Consistently, the results of GSEA indicated that the up-regulated pathways in DDR C2 were as follows: B cell receptor signaling pathway, cytokine-cytokine receptor interaction, JAK-STAT signaling pathway, natural killer cell mediated cytotoxicity, and Notch signaling pathway (**Figure 7C**). DDR C1 was characterized by the DNA replication, gluconeogenesis, lysosome, oxidative phosphorylation, and RNA degradation (**Figure 7D**). These results highlight the distinct molecular functions and pathways between subtype1 and subtype2.

### Identification of the immune characteristics between DDR subtypes

**Figure 8:**
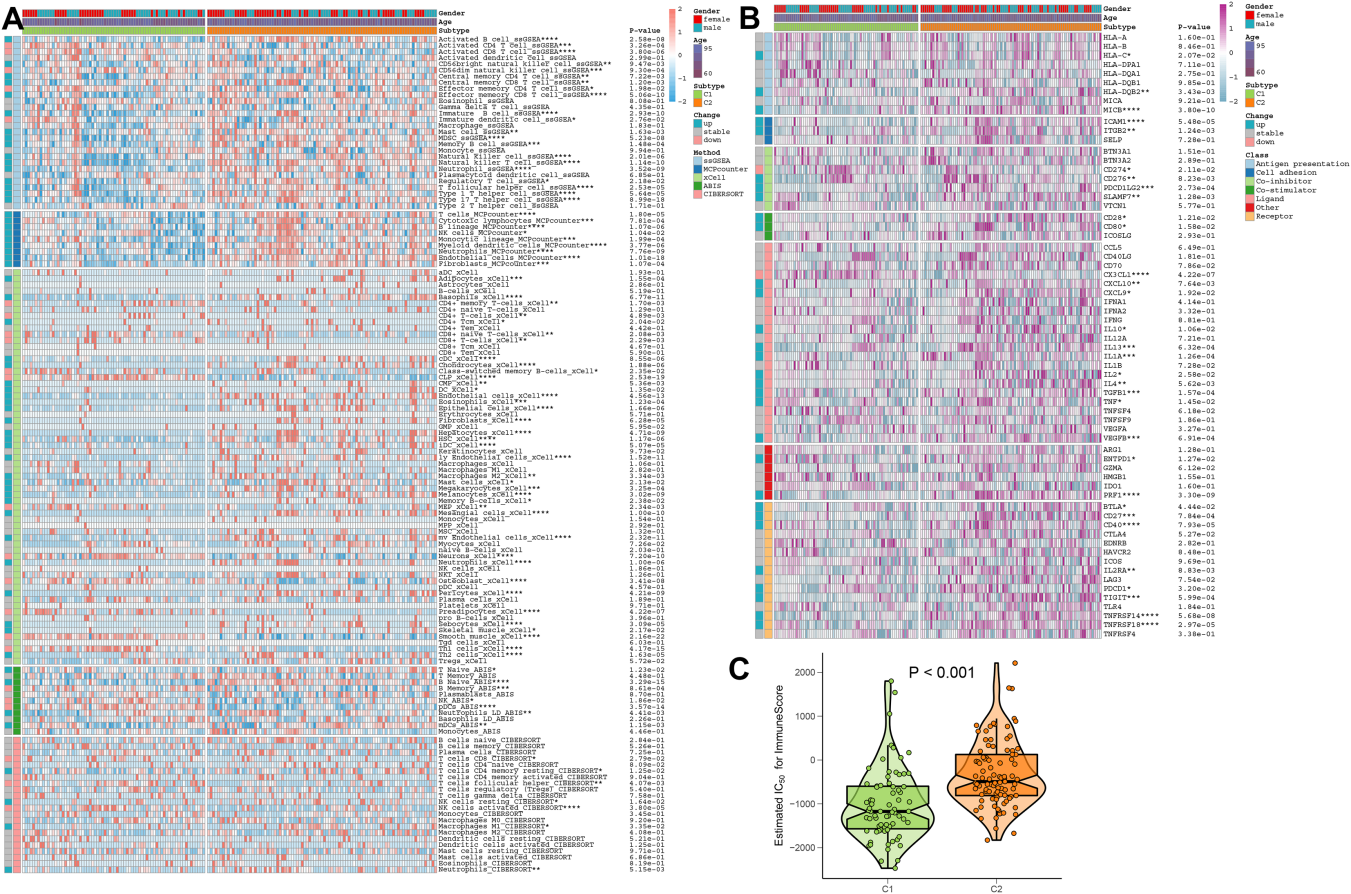
The immunological characteristics of different DDR subtypes. **(A)** Heatmap displaying the expression profiles of infiltrating immune cells across DDR C1 and C2 subtypes in AD patients based on the ssGSEA, MCPcounter, xCell, ABIS, and ESTIMATE algorithms. Age and gender are displayed as patient annotations. *p < 0.05, **p < 0.01, ***p < 0.001, ****p < 0.0001. **(B)** Heatmap displaying the expression profiles of immunoregulatory subgroup genes in DDR C1 and C2 patients with AD. Age and gender are displayed as patient annotations. *p < 0.05, **p < 0.01, ***p < 0.001, ****p < 0.0001. **(C)** A comparison of the immunological score between AD patients with DDR C1 and C2 subtypes.

To better elucidate the differences in immune microenvironment between DDR subtypes, we first comprehensively investigated the correlations between immune infiltrating levels. The heatmap of immune cell subsets based on the ssGSEA, MCPcounter, xCell, ABIS, and ESTIMATE algorithms method was illustrated in **Figure 8A**, which showed that the infiltrating levels of most types of immune cells were primarily distributed in DDR C2, such as B cells, T cells, dendritic cells, macrophages, natural killer cells, and neutrophils. Next, we further investigated the correlations between DDR phenotypes and various immune modulators and immune checkpoints. The results of the effect of DDR on the AD immune microenvironment revealed that the expression levels of co-stimulators, such as CD28 and CD80, in DDR C2 subtype was significantly increased. In addition, the increasing expressions of antigen presentation, cell adhesion, co-inhibitor, ligand, receptor, and other functions were also seen in DDR C2 subtype. While compared to DDR C2, DDR C1 exhibited the higher expression of CD274 and CX3CL1 (**Figure 8B**). Moreover, DDR C2 also exhibited a stronger ImmuneScore, which suggested a more powerful responsiveness to immunotherapy (**Figure 8C**). Based on the above results, we thought of DDR C2 as a subtype of the immune system and DDR C1 as a phenotype of metabolism.

### Machine learning-based selection of characteristic DDR-associated lncRNAs

**Figure 9:**
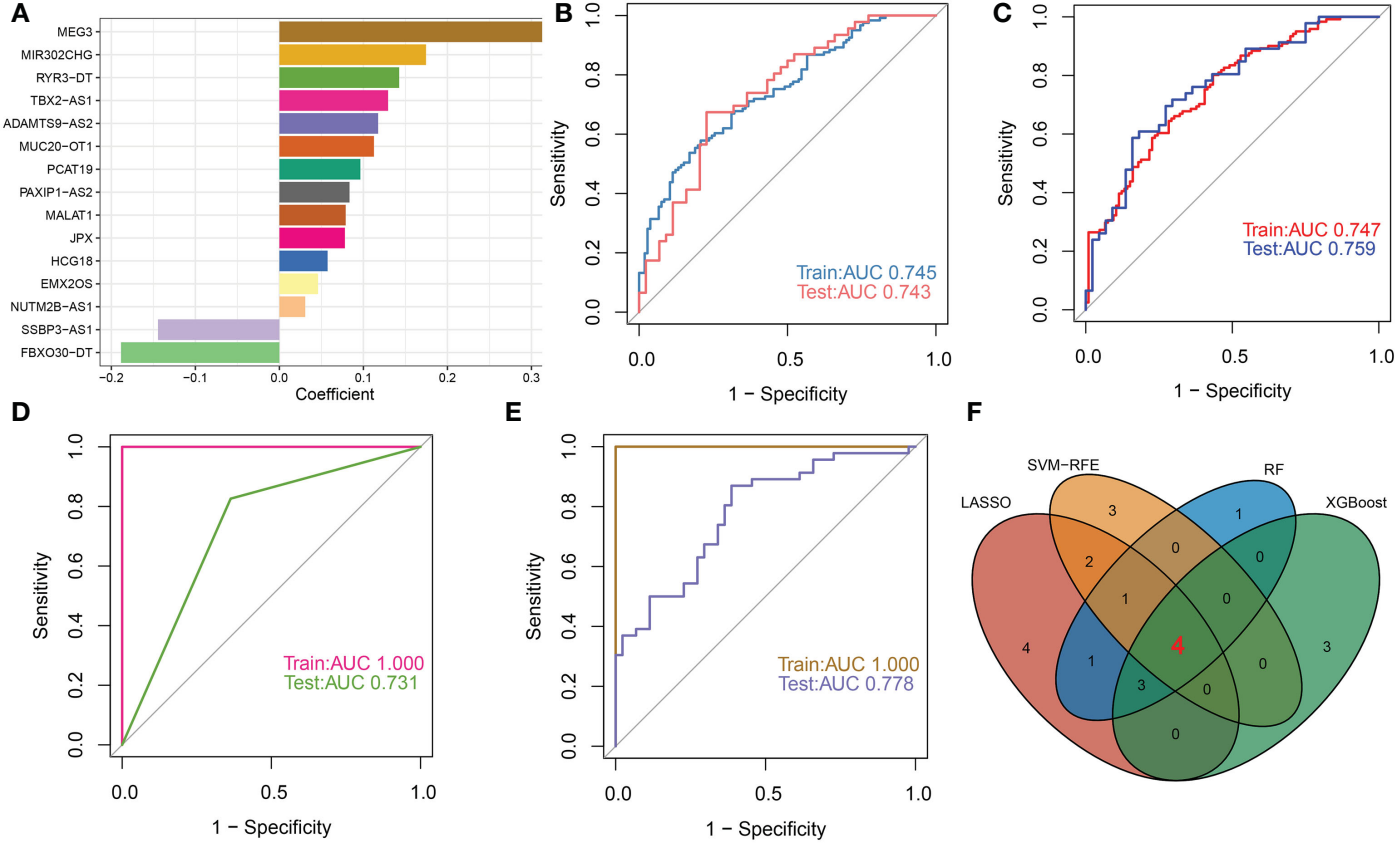
Selection of characteristic lncRNAs associated with DDR based on multiple machine learning models. **(A)** The specific coefficient value of the 15 DDR-associated lncRNAs screened by the optimal lambda value using the LASSO algorithm. **(B)** The value of ROC curves for the 15-lncRNA-based LASSO algorithm in the training set and testing sets. **(C)** The value of ROC curves for the 9-lncRNA-based SVM-RFE algorithm in the training set and testing sets. **(D)** The value of ROC curves for the 14-lncRNA-based RF algorithm in the training set and testing sets. **(E)** The value of ROC curves for the XGBoost algorithm in the training set and testing sets. **(F)** Venn diagram displaying the intersection results of the LASSO, SVM-RFE, RF and XGBoost algorithms.

To further identify DDR-associated lncRNAs that can accurately diagnose AD, we utilized four well-known machine learning models, including LASSO, RF, SVM-RFE, and XGBoost to perform signature selection on the basis of the expression landscape of 33 DDR-associated lncRNAs. Using the LASSO classifier, the best lambda value of 0.0237 was fitted to the LASSO model, which proved to be the most accurate (Additional file: **Figure S5A, B**), eventually generating 15 DDR-associated lncRNAs with non-zero coefficients (**Figure 9A**). ROC curve analysis revealed that the AUC of the 15-lncRNA-based LASSO model was 0.745 in the training set and 0.743 in the test set (**Figure 9B**). Subsequently, we determined that the combination of 9 lncRNAs exhibited the highest accuracy in predicting AD initiation based on the SVM-RFE model (Additional file: **Figure S5C**), with a satisfactory AUC value in the training set (0.747) and the test set (0.759), respectively (**Figure 9C**). Next, we employed the Boruta feature selection algorithm and identified 14 tentative and important lncRNAs in total (Additional file: **Figure S5D**). These 14 signatures screened by the Boruta algorithm were incorporated into the RF model and achieved an AUC value of 1 in the training set and 0.731 in the test set (**Figure 9D**). The top ten most important lncRNAs associated with the RF model were determined according to the feature importance ranking (MALAT1, TBX2-AS1, MEG3, HCG18, JPX, PAXIP1-AS2, MUC20-OT1, FBXO30-DT, DAMTS9-AS2, and MIR23AHG) (Additional file: **Figure S5E**). Furthermore, for AD identification, the XGBoost classifier achieved AUCs of 1 and 0.778 in the training set and test set, respectively (**Figure 9E**). According to the weighted ranking of characteristic DDR-associated lncRNAs in the SHAP dependent analysis of the XGBoost model, the 10 lncRNAs that contributed the most to the XGBoost model were as follows: MALAT1, TBX2-AS1, MEG3, FBXO30-DT, JPX, DAPK1-IT1, MIR570HG, PAXIP1-AS2, SEPSECS-AS1, and ADAMTS9-AS2 (Additional file: **Figure S5F**). The higher the SHAP score of the DDR-associated lncRNAs, the higher the probability of AD. For example, in the XGBoost model, low MALAT1 feature values ​​correspond to inferior SHAP values ​​and are negatively associated with AD occurrence. In contrast, high feature values for MALAT1 resulted in positive SHAP values and an increment in AD risk. For more details on these 10 lncRNAs affecting XGBoost model predictions, see (Additional file: **Figure S5G**). Following intersection, FBXO30-DT, ADAMTS9-AS2, TBX2-AS1, and MEG3 shared by the LASSO, SVM-RFE, RF, and XGBoost algorithms were determined as the common DDR-associated lncRNAs (**Figure 9F**).

### Establishment of a riskScore model and nomogram

**Figure 10:**
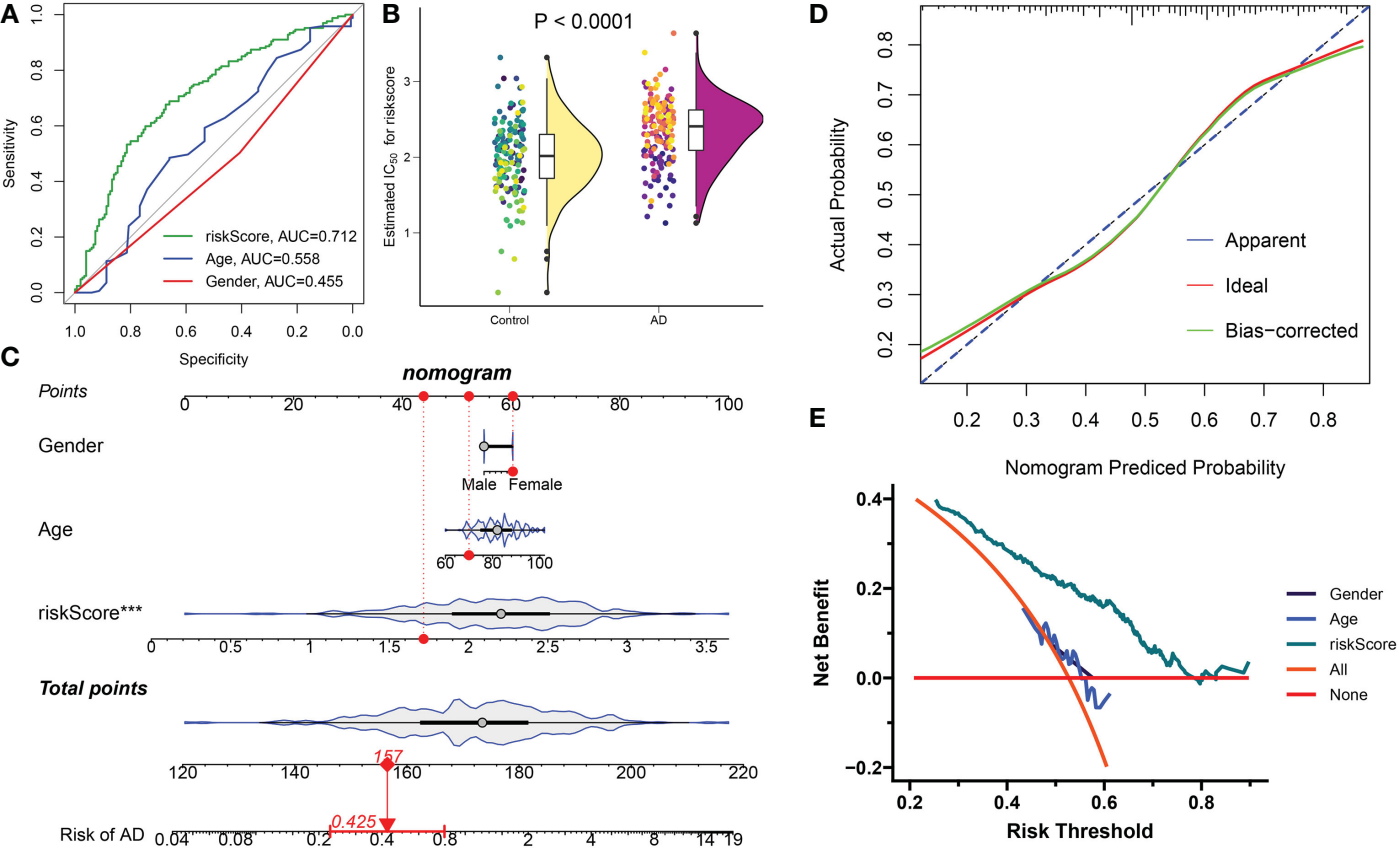
Construction and validation the diagnostic efficacy of riskScore. **(A)** ROC curves displaying the diagnostic efficacy of riskScore and classic clinical indicators in the combined dataset. **(B)** Comparison of riskScore across AD and normal brain tissues in the combined dataset. **(C)** Establishment of a predictive nomogram consisting of riskScore, age, and gender. **(D)** Calibration curve displaying the predicted efficacy of a predictive nomogram. **(E)** DCA showing the clinical benefits of a predictive nomogram.

These four distinct DDR-associated lncRNAs were used to calculate DDR-related riskScore using their corresponding LASSO model coefficients: riskScore = (-0.188478 × FBXO30-DT) + (0.117249 × ADAMTS9-AS2) + (0.129318 × TBX2-AS1) + (0.320641 ×MEG3). Subsequently, the diagnostic efficacy of riskScore and clinical characteristics (age and gender) in predicting AD progression in the combined dataset was estimated using ROC curve analysis. The ROC curve-based prediction model had an AUC value of 0.712, an age of 0.558, and a gender of 0.455, with AD patients exhibiting a significantly higher riskScore (**Figures 10A, B**). These results suggested that the riskScore can predict AD progression more accurately than classic clinical indicators. A constructed nomogram consisting of gender, riskScore, and age demonstrated the satisfactory prediction outcomes of these features in diagnosing AD (**Figure 10C**), and the calculated calibration curve proved the robustness of our nomogram (**Figure 10D**). In addition, the DCA demonstrated the riskScore-based nomogram to be clinically beneficial (**Figure 10E**).

### Clinical values and molecular pathways in the low- and high- risk groups

**Figure 11:**
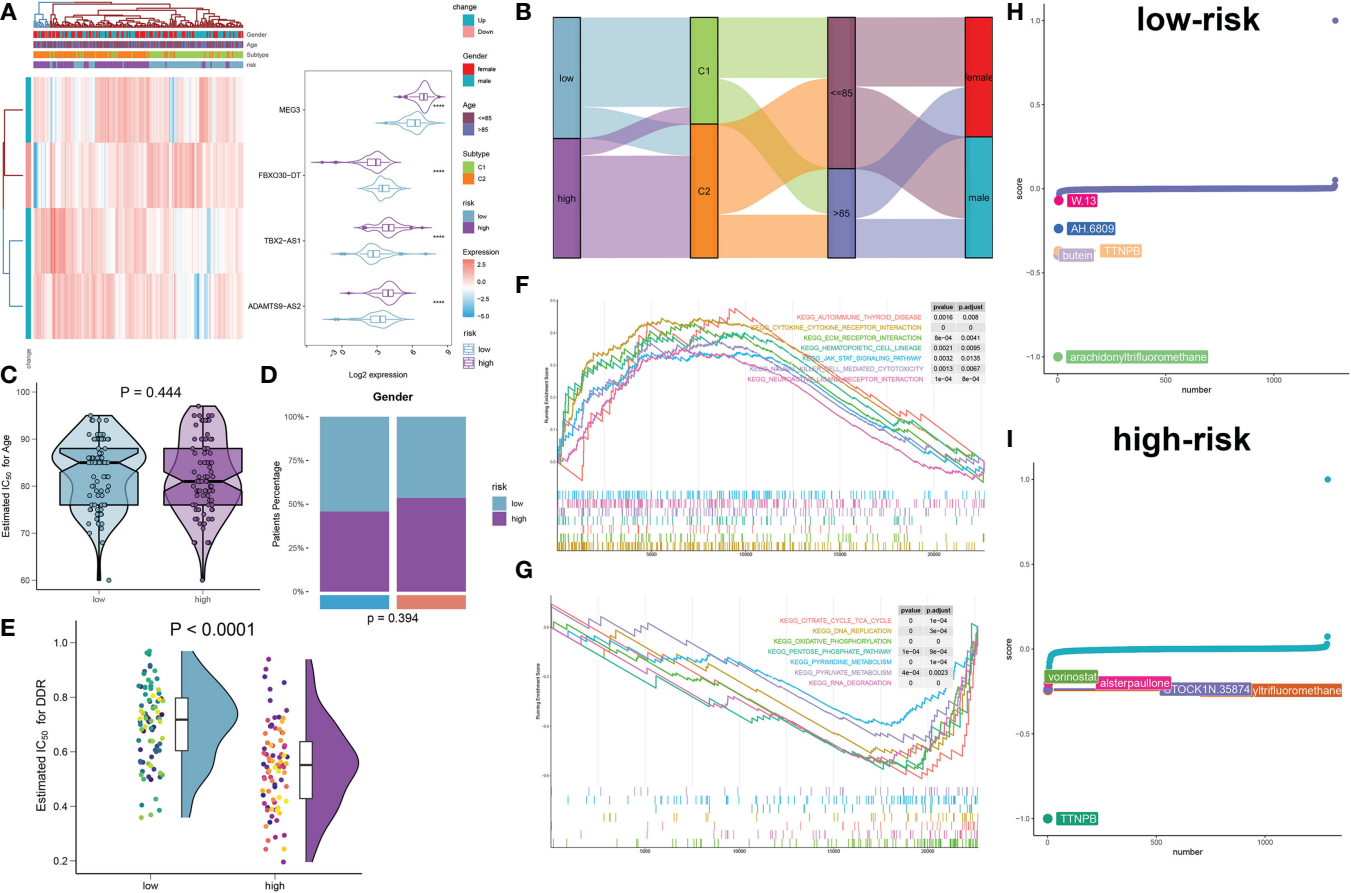
Construction and molecular characteristic of a risk model. **(A)** Heatmap displaying the expression profiles of 4 characteristic lncRNAs associated with DDR between low- and high-risk AD patients. Age, sex, and DDR subtypes were exhibited as patient annotations. **(B)** The sankey diagram illustrating the association between riskScore, DDR subtypes, age, and gender. **(C, D)** Age **(C)** and gender **(D)** distribution differences between AD patients at low and high risk. **(E)** Comparison of the DDR score in low- and high-risk AD patients. **(F, G)** GSEA revealing the elevated **(F)** and downregulated **(G)** main pathways in patients at low and high risk. **(H, I)** CMap study displaying the top 5 prospective treatment medicines for patients at low **(H)** and high **(I)** risk.

To comprehensively illustrate the DDR riskScore-related mechanisms in AD, we developed a risk model and classified 167 AD samples in total into low- and high-risk groups based on their median riskScore. The heatmap and violin plot exhibited the different expression patterns of these four DDR-associated lncRNAs between the low- and high-risk groups, with the reinforced expression levels of MEG3, TBX2-AS1, and ADAMTS9-AS2 in the high-risk group. Whereas a notable higher expression of FBXO30-DT was observed in the low-risk group (**Figure 11A**). The Sankey plot comprehensively depicted details of the different subtypes and proportions of clinical indicators in the low- and high risk groups. Higher DDR C2 subtype rates was found in the high-risk group, while gender and age distribution had no obvious difference between low- and high-risk groups (**Figures 11B–D**). Furthermore, by comparing DDR score levels between these two riskScore groups, it was shown that the DDR score was higher in the low-risk group relative to the high-risk group (**Figure 11E**). GSEA revealed that the high-risk group was predominantly participated in autoimmune disease, cytokine-cytokine receptor interaction, ECM receptor interaction, JAK-STAT signaling pathway, natural killer cell-mediated cytotoxicity, and neuroactive ligand receptor interaction (**Figure 11F**), while low-risk group was primarily regulated by the pathways associated with TCA cycle, DNA replication, oxidative phosphorylation, pentose phosphate pathway, pyrimidine and pyruvate metabolism, and RNA degradation (**Figure 11G**). To evaluate individualized clinical treatments for AD patients, we explored potential therapeutic agents for low- and high-risk populations separately using the CMap database. The top 5 drugs harboring individualized therapeutic potential for the low-risk group were as follows: W-13, XAH-6809, TTNPB, butein, and arachidonyltrifluoromethane (**Figure 11H**). While vorinostat, alsterpaullone, STOCKIN-35874, arachidonyltrifluoromethane, and TTNPB were the five most effective therapeutic drugs for AD patients at high risk (**Figure 11I**). In particular, arachidonyltrifluoromethane and TTNPB had the lowest CMap scores in the low- and high-risk groups, respectively, revealing the best therapeutic benefit in AD patients at different risks.

### Differences in the immune microenvironment and therapeutic drugs between distinct AD risk patients

**Figure 12:**
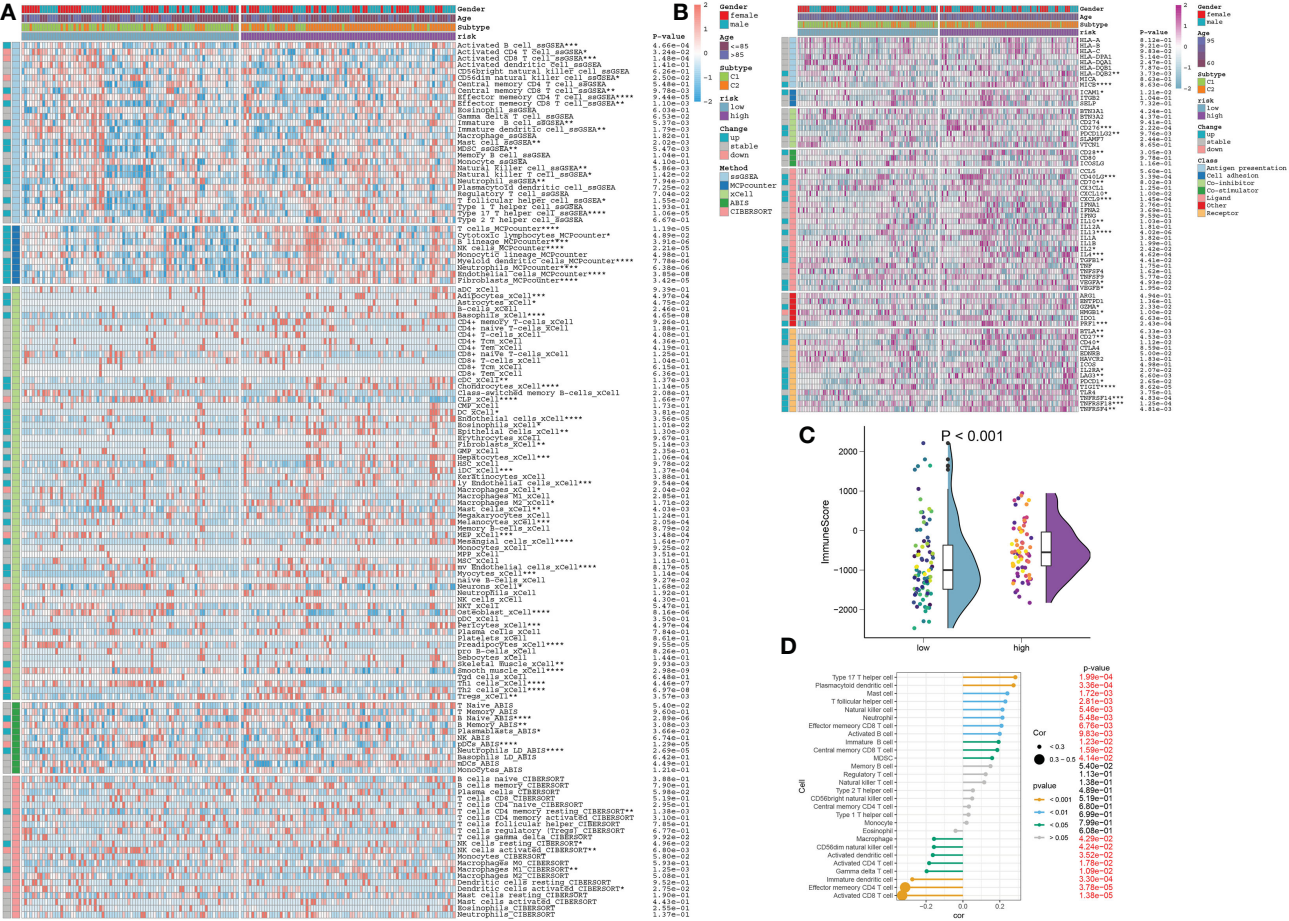
The immunological characteristics in AD patients at low and high risk. **(A)** Heatmap displaying the expression profiles of infiltrating immune cells between low- and high-risk AD patients based on the ssGSEA, MCPcounter, xCell, ABIS, and ESTIMATE algorithms. Age, gender, and DDR subtypes are displayed as patient annotations. *p < 0.05, **p < 0.01, ***p < 0.001, ****p < 0.0001. **(B)** Heatmap displaying the expression profiles of immunoregulatory subgroup genes in AD patients at low and high risk. Age, gender, and DDR subtypes are displayed as patient annotations. *p < 0.05, **p < 0.01, ***p < 0.001, ****p < 0.0001. **(C)** Comparison of the immunological score in low- and high-risk AD patients. **(D)** The correlation between 28 immune cell subtypes and the riskScore.

The landscape of infiltrating cells in low- and high-risk groups was also explored based on the ssGSEA, MCPcounter, xCell, ABIS, and ESTIMATE algorithms. Higher infiltration levels of multiple immune cell subtypes, such as B cells, T cells, macrophages, natural killer cells, and neutrophils were observed in the high-risk group (**Figure 12A**). Moreover, immune-modulators and immune checkpoints differ significantly between patients at different risk for AD. For example, immune genes associated with antigen presentation (HLA-DQB2 and MICB), cell adhesion (ICAM1), co-inhibitor (CD276 and PDCD1LG2), co-stimulator (CD28), ligand (CD40LG, CD70, CXCL10, CXCL9, IL10, IL13, IL2, IL4, TGFB1, VEGFA, and VEGFB), receptor (BTLA, CD27, CD40, IL2RA, LAG3, PDCD1, TIGIT, TNFRSF14, TNFRSF18, and TNFRSF4) and other immune-modulators (GZMA and PRF1) were markedly higher in high-risk group. In contrast, low-risk patients only displayed excessive expression of HMGB1 relative to high-risk patients with AD (**Figure 12B**). Additionally, a significant weakness of ImmuneScore could be observed in the low-risk group, suggesting a poor responsiveness to immunotherapy (**Figure 12C**). Correlation analysis also demonstrated that a higher riskScore was positively correlated with most types of immune cells and revealed a superior infiltration levels of immune (**Figure 12D**).

## Discussion

Currently, due to the heterogeneity of AD, patients exhibit distinct response rates and clinical outcomes, and little progress has been made in individualizing AD therapy. Therefore, comprehensively elucidating the heterogeneity of AD would help us better understand AD pathology and find out more appropriate treatment strategies. Recent research has demonstrated that excessive DNA damage and a defective DNA damage response are closely related to the early phases of AD neuropathology, and that enhanced Aβ production in neural progenitor cells may be the key pathogenic mechanism (12, 13, 41).. According to another study, in stressful circumstances, DDR downstream factors promote cell cycle arrest to protect neurons from death, whereas persistent activation of DDR can lead to progressive senescence in surviving neurons and eventually the onset of neurodegenerative disorders (42). These studies suggest that DDR is a prime candidate for enhancing AD prediction, despite the fact that comprehensive multi-omics research on AD are still incredibly uncommon.

Herein, we first systematically explored the relationships between DDR and AD heterogeneity. A total of 179 DDR regulators were enrolled in our study on the basis of previously published literatures, and 51 of which were abnormally expressed in AD patients, demonstrating that the dysregulated DDR might play a crucial role in promoting the initiation of AD. Previous study has reported that phosphorylated p53-induced DDR is markedly reduced in AD brain (41). Consistently, our transcriptomic and single-cell analysis demonstrated a decreased DDR score in patients with AD relative to controls. Interestingly, we innovatively found a lower DDR score was positively associated with the activation of most types of immune cells, including macrophages, memory B cells, effector memory CD8^+^ T cells, central memory CD4^+^ and CD8^+^ T cells, natural killer cells, and neutrophils, pointing out that the interaction of a decreased DDR score with the activation of immune responses may be a novel mechanism contributing to the poor prognosis in AD. Currently, the regulatory role of lncRNAs in DDR has attracted a growing amount of attention. Due to the extensive relationship between lncRNAs and the canonical DDR pathway, DDR-mediated lncRNA expression provides a regulatory mechanism that precisely regulates the expression of DDR-related genes spatially and temporally (43). Mechanistically, DNA damage alters the expression of various lncRNAs, including the regulation of transcription and post-transcription, and RNA degradation (44). On the other hand, lncRNAs are able to alter the expression levels of their target genes through four regulatory modes, including signal, decoy, guide, and scaffold, which in turn directly regulate cellular processes associated with DDR (45, 46). In our current study, we determined a total of 51 DDR-related lncRNAs based on the WGCNA algorithm, all of which exhibited significant differences between AD patients and healthy individuals, suggesting their various roles in patients with AD.

Single-cell analysis is superior in providing insights into the heterogeneity of molecular content and phenotypic characteristics among complex cell populations of different diseases, and has been widely utilized in medical research (47–49). In our transcriptional analysis, the DDR score was shown to be significantly decreased in cognitively impaired patients and was negatively correlated with immune infiltration levels. Therefore, we further conducted single-cell analysis to depict the landscape of DDR score in CI patients. Consistent with transcriptional results, CI samples exhibited a relative lower DDR score. In addition, T cells and B cells in normal samples displayed markedly activated DDR levels compared with other immune cells. Targeting the DDR damage response through enhanced T-cell activation has been reported to be employed in antitumor therapy (50, 51). Thus, we elucidated the relationship between DDR and T cells at the single-cell level, which may partly provide innovative insights for the clinical treatment of AD. Future studies need to further explore the specific role of DDR in different T-cell subsets. On the other hand, we estimated the interaction strength and numbers of immune cells in CI samples, which showed that macrophages, ILC, and pDC cells exhibited notably strong interactions with other cell types in the CI group. Subsequently, the underlying ligand-receptor interactions were further explored, and the stronger intercellular communications, including IL-16-CD4, CD40LG-(ITGAM+ITGB2), CD40LG-(ITGA5+ITGB1), and ANXA1-FPR1 were exhibited in cognitively impaired patients. Overall, our study deeply elucidated that intricate connections within immune cells are a vital factor in the progression of AD.

We have defined two patterns based on 51 co-expressed lncRNAs related to DDR, each exhibited notably different biological functions and pathways, and immune microenvironment. DDR C2 was primarily driven by immune response-related functions and pathways, accompanied by an increment in the proportion of infiltrated immune cells and the expression of immune modulators. Notably, the immune C2 subtype was often related to lower levels of DDR score. Recent studies have demonstrated the close association between DDR and the immune response. Ionizing radiation-induced activation of DDR is the major cause contributing to the interference of immune microenvironment, thus reducing the anti-tumor effect of radioimmunotherapy (52). Moreover, DDR deficiency was considered an important determinant in promoting tumor immunogenicity (53), suggesting that targeting DDR could serve as a potential therapeutic strategy to promote anti-tumor immune responses. In addition, human adenovirus-mediated immune escape is largely associated with the impairment of interferon (IFN) and DDR responses, including the inhibition of the Mre11-Rad50-Nbs1 complex and DNA ligase IV (54). However, whether there are DDR-mediated immune alterations in AD patients is largely unknown. In our current study, we comprehensively estimated the immune profile of DDR C1 and C2 patients, and found most types of immune cells were primarily distributed in DDR C2, including B cells, T cells, dendritic cells, macrophages, natural killer cells, and neutrophils. These innate and adaptive immune cells play a crucial role in promoting the over-accumulation of amyloid beta, the initiation of tau pathology, and neuroinflammation, eventually leading to the AD progression (55–57). Furthermore, multiple types of immune modulators, including antigen presentation, cell adhesion, co-inhibitor, co-stimulator, ligand, and receptor-related genes were also predominant in DDR C2. It has been reported that human leukocyte antigen (HLA) super families are key members involved in adaptive immunity and enable to trigger the initiation of AD-like neuropathy through activating antigen presentation (58, 59). Other classical immune checkpoint inhibitors, ICAM1, CCL5, and CTLA-4, are also targets of immunotherapy (60–62), suggesting their great therapeutic potential in diseases. Overall, we innovatively defined DDR C2 as an immune subtype, suggesting that immunotherapy targeting DDR could achieve superior efficacy in AD patients with the C1 subtype.

We identified a 4-DDR-related lncRNA signature based on the multiple machine learning algorithms, including FBXO30-DT, TBX2-AS1, ADAMTS9-AS2, and MEG3. As the newest member of the lncRNA family, the role of FBXO30-DT in disease progression remains unknown. Bioinformatics analysis and an external validation experiment revealed that TBX2-AS1 is a member of the M2 tumor-associated macrophages-associated gene family, and the dysregulated expression of TBX2-AS1 could be observed in ovarian cancer, indicating a significant correlation between TBX2-AS1 and the prognostic survival of patients with ovarian cancer (63). ADAMTS9-AS2 is identified as the novel tumor suppressor and could serve as a tumor biomarker in non-small cell lung cancer (64). The upregulated ADAMTS9-AS2 functions as a competing endogenous RNA for miR-143-3p that pretects ITGA6 from miRNA-mediated degradation, thereby promoting the metastasis of salivary adenoid cystic carcinoma by activating the PI3K/Akt and MEK/Erk signaling pathways (65). In contrast, the deficiency of ADAMTS9-AS2 is positively correlated with poor overall survival in patients with ovarian cancer by elevating the proliferation and invasion of tumor cells (66). However, it is regrettable that the relationship between these three characteristic lncRNAs and the pathological progress of AD has not been reported. Interestingly, MEG3 overexpression exacerbates cerebral ischemia-reperfusion injury, but improves cognitive impairment and alleviates pathological damage in AD patients (67, 68). In our current study, the constructed ROC curves, nomograms, calibration curves, and DCA demonstrated that the riskScore based on the 4-DDR-related lncRNA signature can predict AD progression more precisely than individual variable. In total, these findings indicated that the upregulation of a 4-DDR-related lncRNA signature might be closely linked to a poor prognosis in AD patients.

We therefore divided patients with AD into low- and high-risk groups based on the constructed riskScore. Similarly, the high-risk group was defined as the immune phenotype, corresponding to a higher rate of DDR C2 subtype and lower levels of DDR score, while the low-risk group exhibited the opposite effect. Low levels of DDR score in the high-risk group may imply a weaker capacity of DNA repair, which was positively correlated with the increase of the immune responses-mediated pathway. The increment of immune score, infiltrated immune cells, and immune modulators suggesting that high-risk immune phenotype could benefit strongly from immunotherapy. In addition, we further estimated the potential therapeutic drugs targeting AD patients with different risks using the CMap analysis and found that arachidonyltrifluoromethane and TTNPB may exert most effective therapeutic efficacy for AD patients in low-risk and high-risk groups, respectively. As a retinoic acid mimetic, TTNPB has been shown to promote neuronal differentiation *via* reinforcing the activities of RARα and RARγ, thus exerting neuroprotective effects (69). Arachidonyltrifluoromethane, a cPLA2 inhibitor, plays a vital role in attenuating lysosomal membrane permeabilization, inhibition of autophagy, and neuron death, eventually providing neuroprotective and anti-neuroinflammatory effects (70). In our study, the developed risk model not only aids in the implementation of immunotherapy, but also serves as a critical reference for individualized treatment and precision medicine in Alzheimer’s disease patients.

To the best of our knowledge, we were the first to comprehensively assess DDR expression patterns in Alzheimer’s disease patients. However, several limitations need to be emphasized: 1) The current research is retrospective, and only a limited sample size could be obtained from public databases. Further multicenter prospective studies need to be carried out to verify our results. 2) The expression landscape of mRNA was based on microarray datasets, and the results were not as stable as those from *in vivo* or *in vitro* experiments. 3) Larger AD sample sizes with more prognostic and therapeutic information should be taken into account to determine the clinical utility of AD patients with distinct molecular subtypes and riskScore.

## Conclusions

In conclusion, our research showed that the progression of AD and DDR regulators are tightly related. Additionally, we discussed the DDR levels in AD patients at the single-cell level. Furthermore, based on the lncRNAs related with DDR, we developed a unique molecular classification. Different DDR expression patterns, biological traits, and immunological characteristics were exhibited in two subtypes, and the DDR C2 subtype may respond favorably to immunotherapy. Furthermore, we developed a DDR-related risk model to precisely predict the clinical outcomes of AD patients based on the 4-DDR-related lncRNA signature (FBXO30-DT, TBX2-AS1, ADAMTS9-AS2, and MEG3) screened by the various machine learning algorithms. Finally, it was determined that arachidonyltrifluoromethane and TTNPB were promising treatments for AD patients with low and high risk, respectively. Overall, our findings facilitate to design individualized treatments for AD patients and offer new insights into the heterogeneity of AD based on DDR regulators.

## Data availability statement

The original contributions presented in the study are included in the article/**Supplementary Material**. Further inquiries can be directed to the corresponding authors.

## Author contributions

Experimental design: CL, FL, and YL; Gathering and processing of data: MC, HL, XL, LW, and YZ; Initial drafts of the writing: YL, XL, and MC; Manuscript revision: CL, FL, and HL. All authors contributed to the article and approved the submitted version.

## Glossary

**Table:**

AD	Alzheimer’s disease
DDR	DNA damage response
lncRNA	Long non-coding RNAs
CI	cognitive impairment
CSF	Cerebrospinal fluid
WGCNA	Weighted gene coexpression network analysis
DEGs	Differential expressed genes
XGBoost	eXtreme Gradient Boosting
RF	Random Forest
LASSO	least absolute shrinkage and selection operator
SVM-RFE	Support vector machine-recursive feature elimination
RMA	Robust Multiple Array Average
PCA	Principal component analysis\
UMAP	Uniform manifold approximation and projection
GEO	Gene Expression Omnibus
ssGSEA	single sample gene set enrichment analysis
TOM	topological overlap matrix
CDF	Cumulative distribution function
GO	Gene ontology
KEGG	Kyoto Encyclopedia of Genes and Genomes
BP	biological processes
MF	molecular functions
CC	cellular components
GSVA	Gene set enrichment Analysis
MsigDB	Molecular Signatures Database
GSEA	Gene set enrichment analysis
SHAP	Shapley Additive exPlanation
AUC	an area under the curve
DCA	decision curve analysis
CDF	cumulative distribution function
Tsne	t-Distributed Stochastic Neighbor Embedding
Cmap	Connectivity map
