# Supplementary material for: Integration of bulk RNA sequencing and single-cell analysis reveals a global landscape of DNA damage response in the immune environment of Alzheimer’s disease
Source: Front Immunol. 2023 Feb 21;14:1115202. doi: 10.3389/fimmu.2023.1115202 (PMC9989175; doi:10.3389/fimmu.2023.1115202)
Supplement: Supplementary file 1 [file DataSheet_1.docx]

**Integration of bulk RNA sequencing and single-cell analysis reveals a global landscape of DNA damage response in the immune environment of Alzheimer's disease**


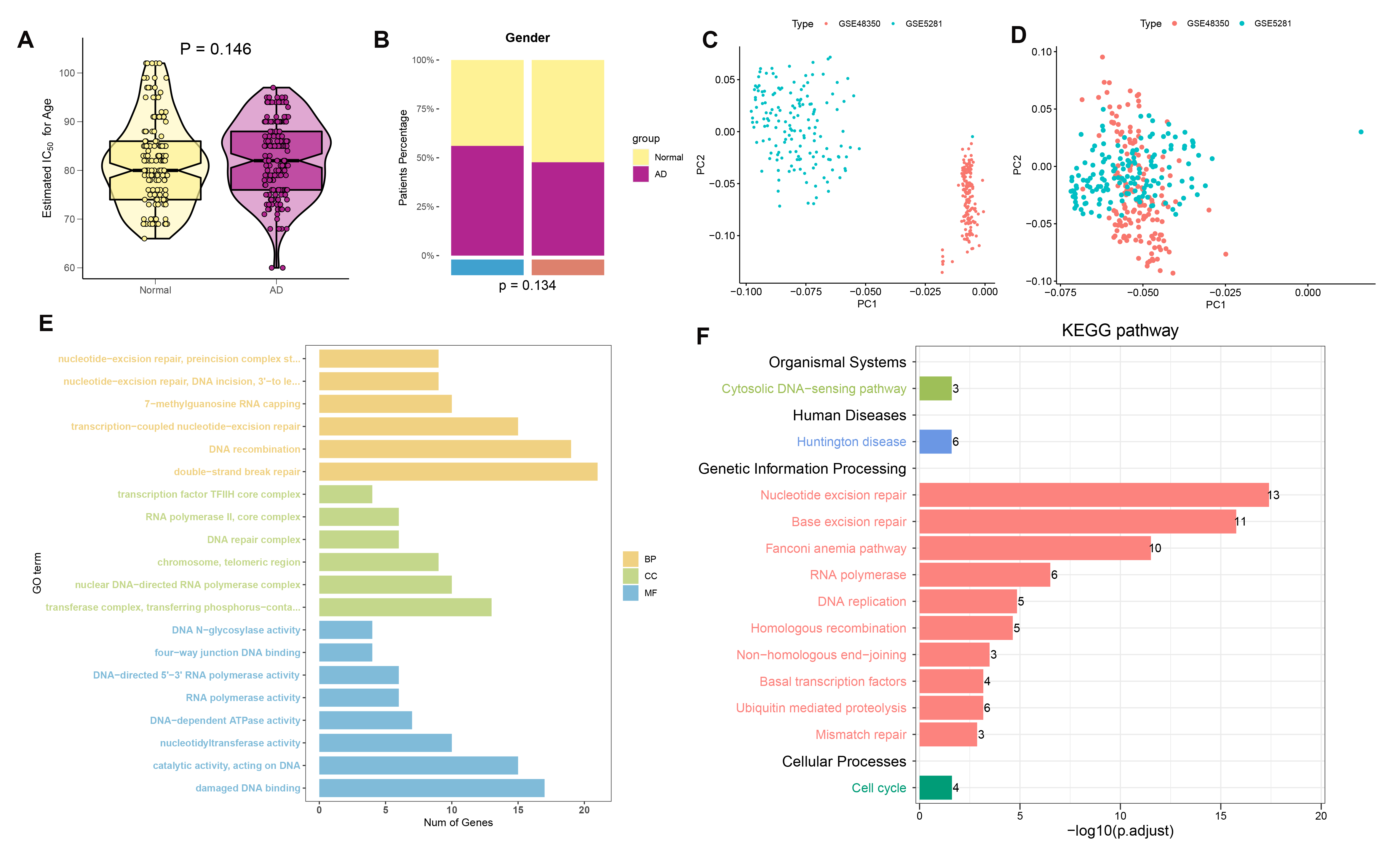


**FigureS1 Evaluation of DDR regulators between AD and healthy individuals**. (A,B) PCA displaying the distribution of samples from GSE48350 and GSE5281 datasets before (A) and after (B) batch correction. Each dot corresponds to one sample and each color corresponds to distinct datasets. (C,D) The differences in the distribution of age and gender between healthy subjects and AD patients were compared in the combined datasets. (E,F) Barplots showing GO (B) and KEGG (C) enrichment analyses of 51 DDR regulators.


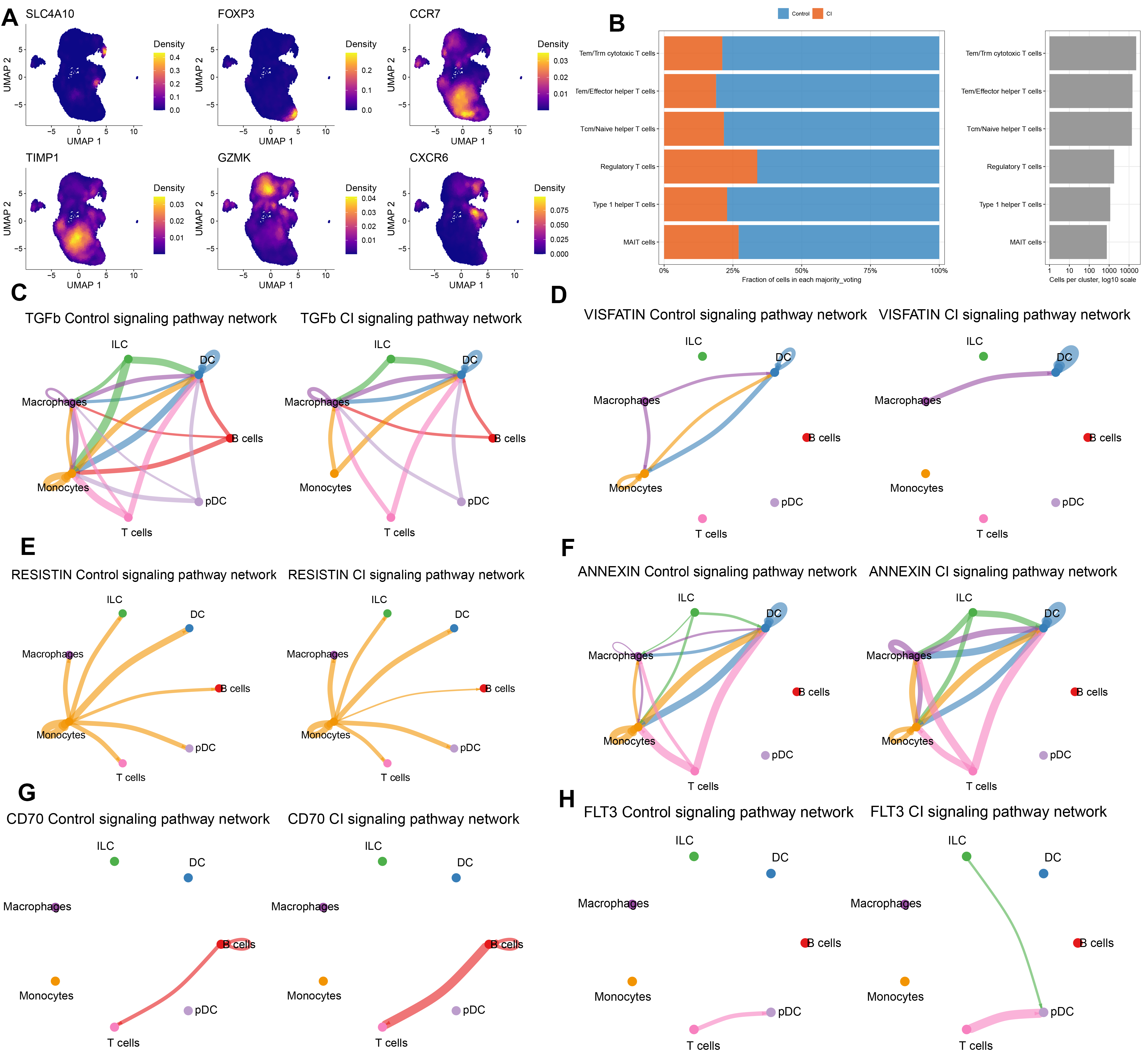


**FigureS2 Subsets of T cells and intercellular communications revealed by single-cell sequencing.** (A) Representative density showing the expression of selected marker genes for MAIT cells, regulatory T cells, Tcm/Naive helper T cells, Tem/Effector helper T cells, Tem/Trm cytotoxic T cells, and Type 1 helper T cells. (B) A stacked bar chart showing the fractions of each T cell subtypes in normal and CI groups, respectively. (C-H) Intercellular pathway difference in TGFb (C), VISFATIN (D), RESISTIN (E), ANNEXIN (F), CD70 (G), and FLT3 signaling pathwayS between normal and CI cognitively impaired CSF samples.


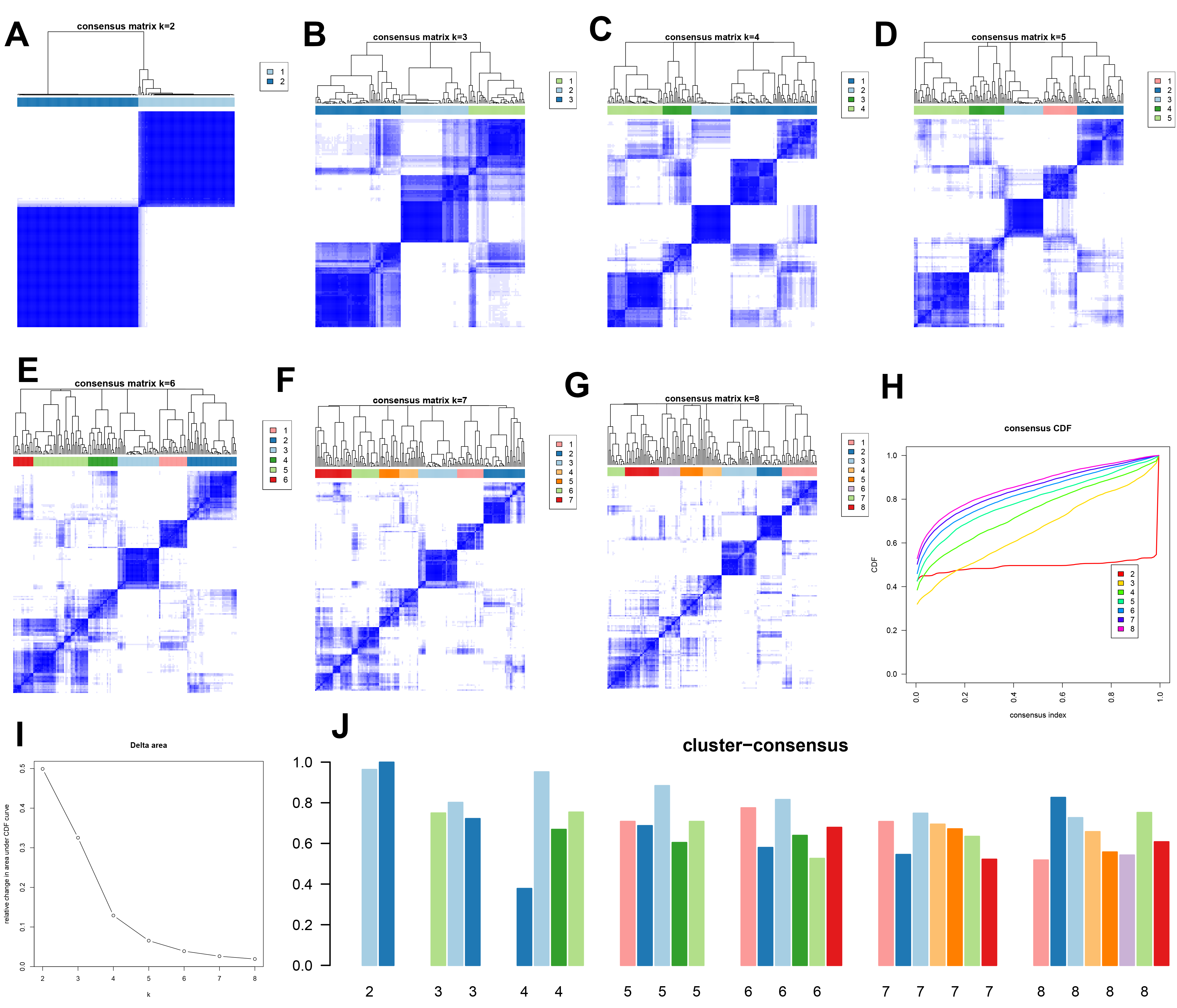


**FigureS3 Identification of DDR subgroups in AD patients using consensus clustering approach in the combined dataset.** (A-G) Consensus clustering matrix for k=2-8. (H) Consensus CDF curves when k=2-8. (I) Relative variations in CDF delta area curves when k=2-8. (J) Consensus score of each DDR subtype when k= 2-8.


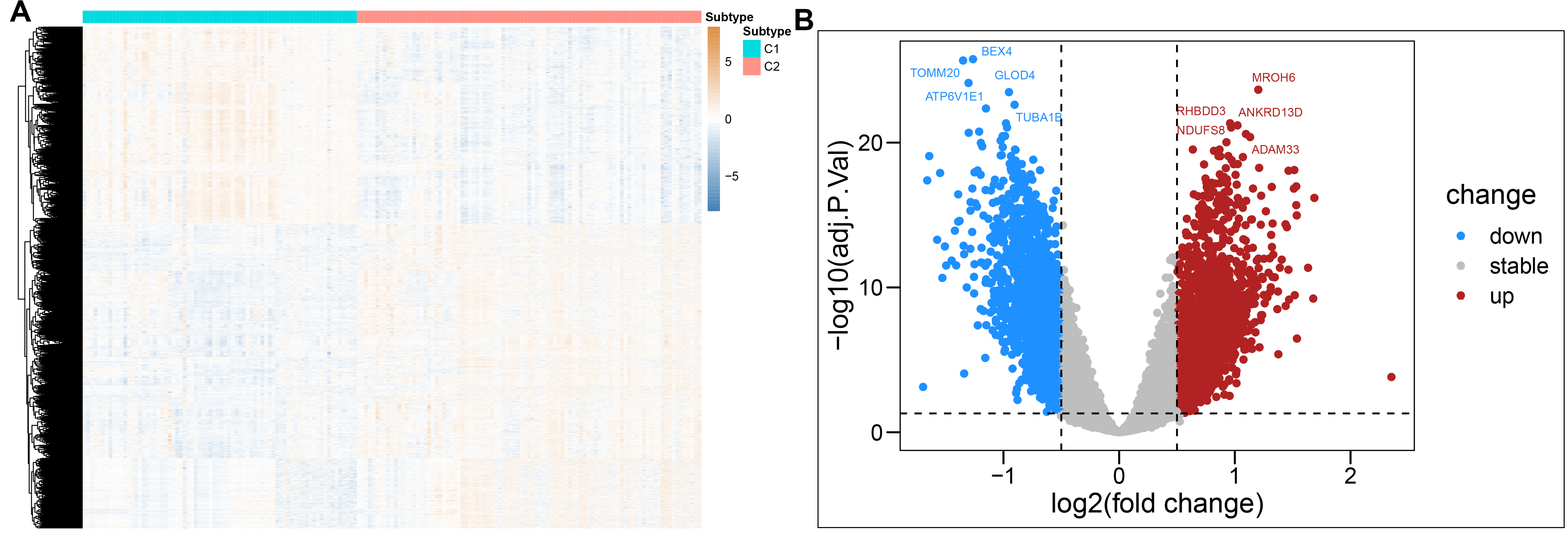


**FigureS4 DEGs screening between DDR C1 and C2 subgroups.** (A,B) Heatmap (A) and volcano plot (B) displaying the expression profiles of elevated and down-regulated DEG.


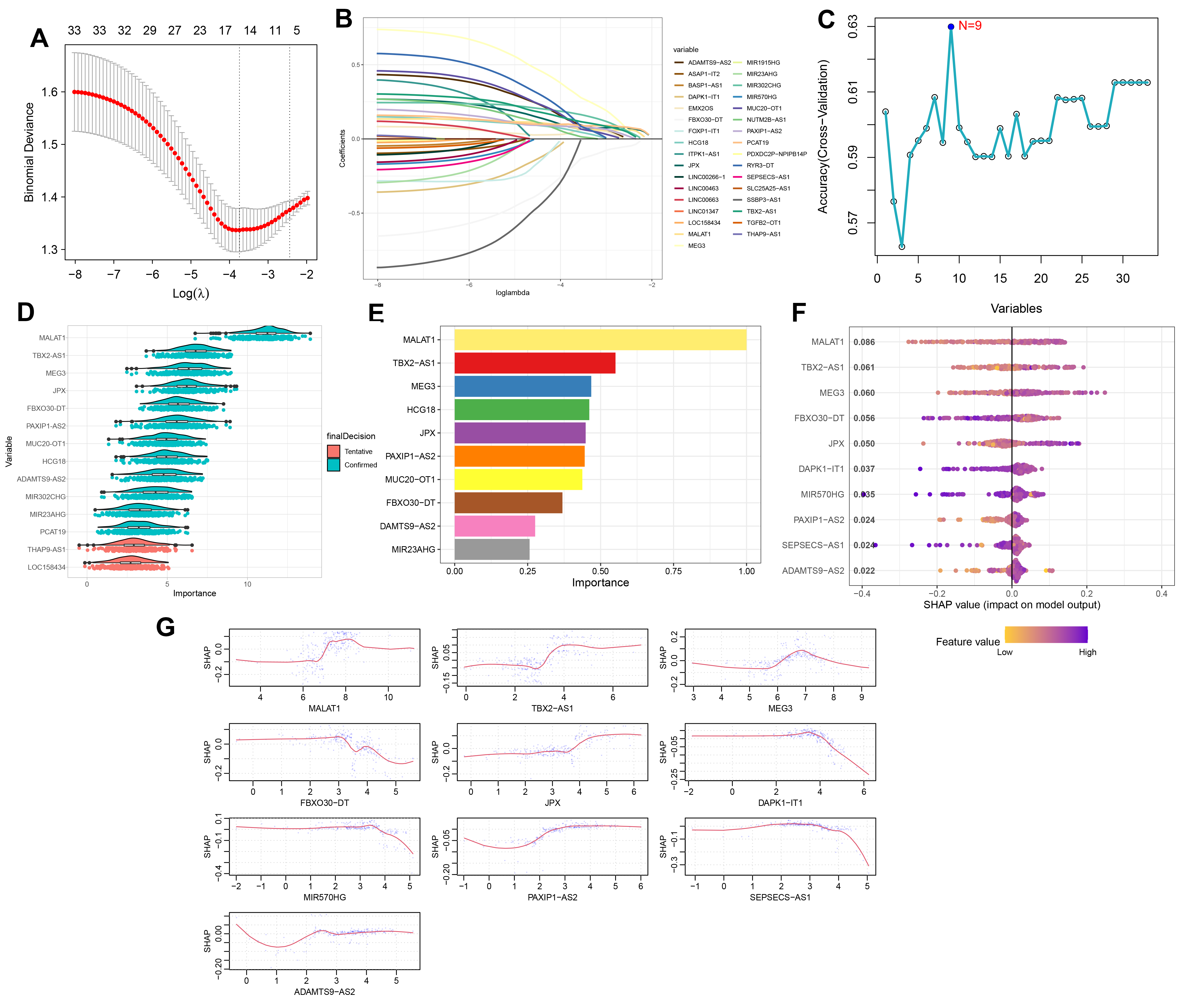


**FigureS5 Selection of characteristic lncRNAs associated with DDR based on multiple machine learning models.** (A) Optimal lambda value selection for the LASSO algorithm based on 10-fold cross-validation. (B) LASSO coefficient landscapes of 33 differentially expressed lncRNAs. (C) The line graph displaying the cross-validated accuracy based on different numbers of lncRNAs in the SVM-RFE model. (D) RainCloud plots showing the importance distribution of 24 DDR-associated lncRNAs screened by the Boruta algorithm. (E) Barplots showing the relative importance rankings of the top 10 DDR-associated lncRNAs based on RF algorithm. (F) SHAP summary plot exhibiting the relative importance rankings of the top 10 DDR-associated lncRNAs contributing to the XGBoost model. (G) SHAP dependence plot displaying the top 10 DDR-associated lncRNAs contributing to the XGBoost model.
